# GATA-targeted compounds modulate cardiac subtype cell differentiation in dual reporter stem cell line

**DOI:** 10.1186/s13287-021-02259-z

**Published:** 2021-03-18

**Authors:** Mika J. Välimäki, Robert S. Leigh, Sini M. Kinnunen, Alexander R. March, Ana Hernández de Sande, Matias Kinnunen, Markku Varjosalo, Merja Heinäniemi, Bogac L. Kaynak, Heikki Ruskoaho

**Affiliations:** 1 Drug Research Program, Division of Pharmacology and Pharmacotherapy, Faculty of Pharmacy, University of Helsinki, P.O. Box 56, FI-00014 Helsinki, Finland; 2 Institute of Biomedicine, School of Medicine, University of Eastern Finland, Kuopio, Finland; 3 Institute of Biotechnology, University of Helsinki, Helsinki, Finland; 4 Helsinki Institute of Life Science, University of Helsinki, Helsinki, Finland

**Keywords:** Stem cells, Cardiomyocyte subtype, Atrial cardiomyocyte, Ventricular cardiomyocyte, Heart regeneration, GATA4, NKX2-5

## Abstract

**Background:**

Pharmacological modulation of cell fate decisions and developmental gene regulatory networks holds promise for the treatment of heart failure. Compounds that target tissue-specific transcription factors could overcome non-specific effects of small molecules and lead to the regeneration of heart muscle following myocardial infarction. Due to cellular heterogeneity in the heart, the activation of gene programs representing specific atrial and ventricular cardiomyocyte subtypes would be highly desirable. Chemical compounds that modulate atrial and ventricular cell fate could be used to improve subtype-specific differentiation of endogenous or exogenously delivered progenitor cells in order to promote cardiac regeneration.

**Methods:**

Transcription factor GATA4-targeted compounds that have previously shown in vivo efficacy in cardiac injury models were tested for stage-specific activation of atrial and ventricular reporter genes in differentiating pluripotent stem cells using a dual reporter assay. Chemically induced gene expression changes were characterized by qRT-PCR, global run-on sequencing (GRO-seq) and immunoblotting, and the network of cooperative proteins of GATA4 and NKX2-5 were further explored by the examination of the GATA4 and NKX2-5 interactome by BioID. Reporter gene assays were conducted to examine combinatorial effects of GATA-targeted compounds and bromodomain and extraterminal domain (BET) inhibition on chamber-specific gene expression.

**Results:**

GATA4-targeted compounds 3i-1000 and 3i-1103 were identified as differential modulators of atrial and ventricular gene expression. More detailed structure-function analysis revealed a distinct subclass of GATA4/NKX2-5 inhibitory compounds with an acetyl lysine-like domain that contributed to ventricular cells (%Myl2-eGFP+). Additionally, BioID analysis indicated broad interaction between GATA4 and BET family of proteins, such as BRD4. This indicated the involvement of epigenetic modulators in the regulation of GATA-dependent transcription. In this line, reporter gene assays with combinatorial treatment of 3i-1000 and the BET bromodomain inhibitor (+)-JQ1 demonstrated the cooperative role of GATA4 and BRD4 in the modulation of chamber-specific cardiac gene expression.

**Conclusions:**

Collectively, these results indicate the potential for therapeutic alteration of cell fate decisions and pathological gene regulatory networks by GATA4-targeted compounds modulating chamber-specific transcriptional programs in multipotent cardiac progenitor cells and cardiomyocytes. The compound scaffolds described within this study could be used to develop regenerative strategies for myocardial regeneration.

**Supplementary Information:**

The online version contains supplementary material available at 10.1186/s13287-021-02259-z.

## Introduction

Myocardial infarction results in the loss of ventricular heart tissue which is not efficiently replaced [1]. Exogenously delivered pluripotent stem cell-derived cardiac progenitors (CPs) or cardiomyocytes represent a potential cell source for replacement of lost myocardium in the injured adult heart [2, 3]. Additionally, low levels of endogenous cardiomyocyte proliferation occurs via de-differentiation into a progenitor-like state, proliferation of progenitor-like cells, and re-differentiation into mature cardiomyocytes [4, 5]. Importantly, failure of cells to re-differentiate after the induction of de-differentiation and proliferation results in adverse outcomes, including left ventricular hypertrophy, arrhythmias, and sudden death [6, 7]. Thus, small molecules capable of influencing the fate decisions and differentiation programs of multipotent progenitor cells could facilitate therapeutic regeneration of lost myocardium. Indeed, though in vitro expansion and cell transplantation of pluripotent stem cell (PSC) derived CPs, cardiomyocytes, cardiosphere-derived cells, and mesenchymal stem cells are being explored as therapeutic options for cardiovascular diseases [2, 8–16], it is unknown if exogenously delivered CP differentiation could be augmented by simultaneous delivery of chemical inducers of atrial or ventricular cardiomyocyte differentiation. Furthermore, it is unknown whether endogenous progenitors/stromal cells in the adult human heart could be chemically induced to generate functional atrial or ventricular heart muscle to treat adult heart diseases. Additionally, cardiomyocyte subtype differentiation could aid in the generation of stem-cell derived cardiac patches or bioengineered hearts for transplantation [17–19]. Cardiogenic compounds might also modify pathological gene expression programs characterized by activation of fetal gene expression networks, such as those observed during adult heart disease [20, 21].

Though previous in vitro studies have led to the identification of cardiogenic small molecules targeting developmental signaling pathways [22–26], the ubiquitous role of these pathways in non-cardiac organ homeostasis and stem cell niches might limit their use in vivo. This could be circumvented by developing cardiogenic compounds targeting tissue-specific proteins, such as combinations of developmental transcription factors (TFs). Notably, the transition from proliferative multipotent progenitor cells to differentiated cardiomyocytes is orchestrated by core cardiac TFs acting synergistically and antagonistically [27]. These TFs include GATA4/MEF2C/TBX5/NKX2-5, and loss of function phenotypes of these genes demonstrate their effects on cardiac morphogenesis and target gene activation [28–32]. More detailed studies on cardiac TF machinery have revealed a low number of regulatory TFs (e.g., GATA4, HAND2, MEF2, and TBX5) that are required and able to cooperatively reprogram cardiac fibroblasts into functional cardiac-like myocytes in vitro and in vivo [1, 33–35]. Moreover, we have previously identified GATA4 and NKX2-5 as master regulators of stretch-induced hypertrophic responses in differentiated cardiomyocytes [36] and reported the structural basis for the GATA4/NKX2-5 interaction [37]. The observed nuclear receptor-like structure of the GATA4/NKX2-5 complex provides an opportunity for small molecule interference, and we subsequently reported a novel family of compounds targeting the GATA4/NKX2-5 interaction that inhibited synergistic transcription from reporter genes possessing NKX2-5 binding sites [38, 39]. Furthermore, we showed that the hit compound 3i-1000 inhibited cardiomyocyte hypertrophy in vitro and improved left ventricular ejection fraction/structural remodeling after myocardial infarction and other cardiac injuries in vivo [39–41].

Though the importance of GATA4 and NKX2-5 to cardiovascular development and postnatal function has been extensively reported [30, 31, 42], it is unknown to what degree chemical perturbation of GATA4/NKX2-5 synergy affects atrial and ventricular cardiomyocyte differentiation of cardiac progenitors. In the present study, we have tested compounds that we previously identified to inhibit the GATA4/NKX2-5 interaction in a dual reporter assay for the differentiation of atrial and ventricular cardiomyocytes from PSCs, leading to the identification of small molecules modulating atrial and ventricular gene expression, respectively. Chemically induced gene expression changes were characterized by qRT-PCR, global run-on sequencing (GRO-seq) and immunoblotting, revealing the alteration of GATA4 protein and gene regulatory networks by novel compound 3i-1000 during the differentiation process. Structure-function analyses of active compounds implicated the involvement of an acetyl lysine-like fragment that is potentially related to the activity of the bromodomain and extraterminal domain (BET) family of proteins such as BRD4, and GATA4-BRD4 interactions were confirmed by analysis of the GATA4 protein interactome by BioID. Follow-up studies revealed that (+)-JQ1, an inhibitor of BET bromodomains, increased the activity of both GATA4-dependent and GATA4/NKX2-5-dependent chamber-specific transcription programs, and this was inhibited by 3i-1000. Collectively, these experiments resulted in the identification of small molecules 3i-1000 and 3i-1103 as novel selective regulators of atrial and ventricular gene expression, as well as provided insight into the mechanism-of-action of GATA4-targeted compounds involving an acetyl lysine-like subdomain.

## Materials and methods

### Spontaneous and directed differentiation assays of mouse embryonic stem cells

Differentiation assays for dual reporter mouse embryonic stem cells (ESCs) were conducted as described previously [43], though compound treatment windows were modified as indicated. Flow cytometry was performed on a BD Accuri C6 or BD LSRFortessa flow cytometer. Synthesis of compounds used in the present study was performed in the Division of Pharmaceutical Chemistry at the University of Helsinki, Pharmatory (Oulu, Finland), Chembridge (San Diego, USA), and Maybridge (Leicestershire, UK) as described [38, 39]. *All-trans* retinoic acid (ATRA) and (+)-JQ1 were purchased from a commercial provider (Sigma). Compounds were diluted in DMSO prior to administration (final DMSO concentration 0.1% in medium) and values were normalized to DMSO controls. For characterization of chemically differentiated embryoid bodies (EBs) by qRT-PCR, RNA was isolated from D12 EBs using TRIzol reagent (Thermo Fisher Scientific) and RNeasy MinElute Cleanup kit. qRT-PCR reactions were performed using Taqman gene expression assays (Supplementary Table 1), and values were normalized to a reference gene (Actb). qRT-PCR reactions were performed on a Fluidigim Biomark HD system.

### Immunoblotting of GATA4

GATA4 protein was examined in mESC-derived EBs collected on D5 and D12 of differentiation with compound treatments. For overexpression studies, HEK293 cells were transfected with GATA4-V5 tagged plasmid. See extended description of these studies in the Supplementary Methods.

### Analysis of protein sequences and compound conformations

Protein sequences for GATA4 were downloaded from UniProt Knowledgebase (UniProtKB) which contains two separate sections: UniProtKB/Swiss-Prot (SP, manually annotated) and UniProtKB/TrEMBL (TR, computationally annotated). Sequences were aligned by using Clustal Omega (European Bioinformatics Institute, EMBL-EBI).

The commercial modeling package MOE 2019.0102 (Chemical Computing Group Inc., Montreal, Canada; http://www.chemcomp.com) with LowModeMD module was utilized to generate small-molecule conformation databases. A force field MMFF94x suitable for small molecule calculations was applied for molecule parameterizations and energy minimizations as described previously [39]. Moreover, default settings were employed to score and rank conformational databases. The lowest energy conformation was selected as a representative structure of the compound.

### GRO-seq

The GRO-seq method was performed as described previously [44]. Neonatal rat ventricular myocytes (NRVM) were cultured as previously described [39], and isolation of nuclei was performed as detailed in Supplementary Methods. Samples from two biological replicates were pooled so that in GRO-seq analysis there were around 5 M nuclei/sample. A run-on reaction was performed with Br-UTP in the presence of sarkosyl that prevents loading of new polymerases. The run-on products were purified and DNAse treated. Base hydrolysis was used for RNA fragmentation. Anti-BrUTP beads were used to enrich run-on products. Poly-A tailing was used in the first step of sequencing library preparation.

### BioID measurements

Cell line generation for expressing GATA4 or NKX2-5 with N-terminal MAC tag, sample preparation and mass spectrometry was performed similarly as described [45], with the modification of using 1% n-Dodecyl-β-D-Maltoside, instead of 0.5% IGEPAL, during sample preparation. Proteins detected by BioID were filtered using the CRAPome contaminant repository [46].

### Bromodomain assays

Selected compounds (10 μM) were subjected to the Eurofins/DiscoverX BROMOscan™ assay to identify the interaction with biologically relevant bromodomains. In brief, this consists of a cell-free assay in which competitive inhibition is identified based on the interference of the bromodomain interaction under study with a known ligand. In total, test compounds were assayed against 32 bromodomains.

Percent control was calculated as follows:

((test compound signal – positive control signal)/(negative control signal – positive control signal)) × 100.

### Reporter gene assays

COS-1 cells were cultured and reporter gene assays were performed as previously described [40] and in the Supplementary Methods. Each experiment included three replicates. For the analysis of the results, the firefly values were normalized to the vehicle-treated control values, i.e., GATA4 or GATA4/NKX2-5 synergy.

### Data analysis and statistics

Data from differentiation assays, qRT-PCR experiments, and reporter gene assays were analyzed in R. Data were normalized to DMSO (vehicle)-treated and are presented as the mean ± SEM. Differences between compounds- and vehicle-treated groups were determined by performing *T* test or Wilcoxon test as indicated (****P ≤ 0.0001, ***P ≤ 0.001, **P ≤ 0.01, *P ≤ 0.05).

For GRO-seq analysis, the sequencing reads were quality controlled using the FASTX toolbox and mapped to the rat genome using Bowtie. The Homer tool was used to quantify the GRO-seq signal level (http://biowhat.ucsd.edu/homer/chipseq/index.html) in four conditions. Genes were ranked based on their fold change between 2 h treatment and control, and those with at least 1.5-fold change were selected for further analysis based on hierarchical clustering. The library size normalized read counts were visualized as a heatmap. USCS genome browser tracks were generated using Homer to overlay the transcriptional activity data from GRO-seq with gene annotations. For BioID analysis, the proteins detected by mass spectrometry were filtered using the CRAPome contaminant repository [46]. Those proteins seen in more than 10% (41/411) of CRAPome database experiments were discarded.

## Results

### Effects of GATA4-targeted compounds on ventricular cardiomyocyte gene expression during pluripotent stem cell differentiation

**Fig. 1:**
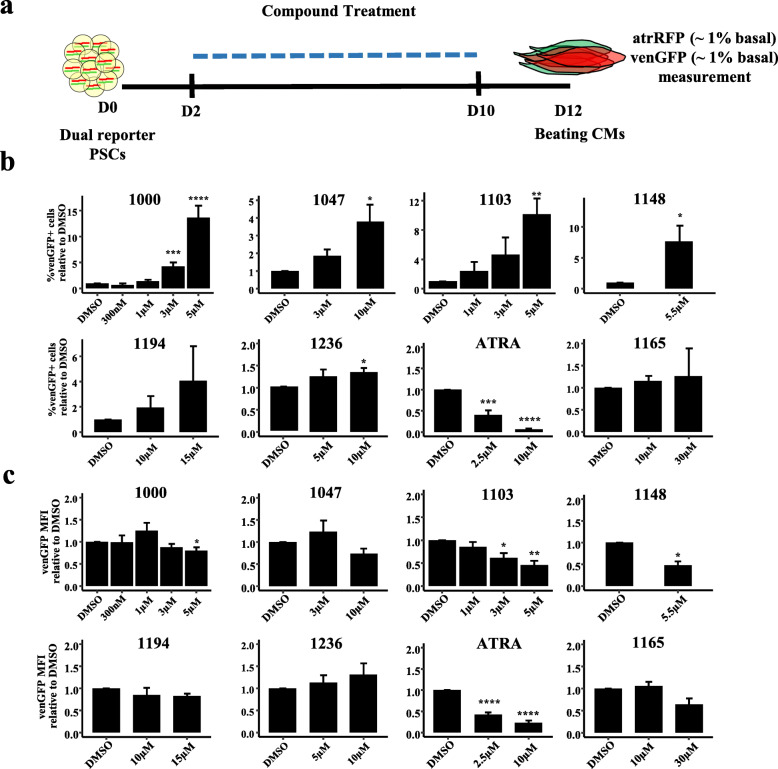
Compound screening for activation of atrial and ventricular reporter genes. **a** Screening strategy for identification of cardiogenic compounds during the spontaneous differentiation of reporter mESCs. GATA4-targeted compounds were screened during a D2–D10 window encompassing mesodermal commitment, cardiac progenitor specification, and differentiation of spontaneously beating cardiomyocytes. Differentiation cultures were measured on D12 of differentiation after treatment with GATA4-targeted compounds for **b** %venGFP+ cells and **c** venGFP-MFI. Pluripotent stem cells (PSCs), cardiomyocyte (CM), atrRFP (SMyHC3-TdTomato, atrial), venGFP (Myl2-eGFP, ventricular), all-trans retinoic acid (ATRA), and mean fluorescent intensity (MFI). Data is presented as mean ± SEM (*n* ≥ 3, independent experiments). ****P ≤ 0.0001, ***P ≤ 0.001, **P ≤ 0.01, *P ≤ 0.05 (*T* test or Wilcoxon vs DMSO control). *n* = 3 (3i-1000 300 nM, 3i-1194 10 μM, 3i-1194 15 μM, 3i-1165 10 μM, 3i-1165 30 μM), *n* = 4 (3i-1103 1 μM, 3i-1236 5 μM, 3i-1236 10 μM), *n* = 5 (3i-1047 3 μM, 3i-1103 3 μM, 3i-1148 5.5 μM), *n* = 7 (3i-1103 5 μM), *n* = 8 (3i-1047 10 μM), *n* = 10 (ATRA 10 μM), *n* = 11 (3i-1000 1 μM), *n* = 13 (ATRA 2.5 μM), *n* = 21 (3i-1000 5 μM), *n* = 26 (3i-1000 3 μM)

In order to test for the effects of GATA4-targeted compounds on differentiation programs of cardiomyocyte subtypes (atrial vs ventricular), a differentiation assay based on the expression of markers of ventricular (Myl2-eGFP, venGFP) and atrial (SMyHC3-TdTomato, atrRFP) cardiomyocytes was used [43]. As compounds affect GATA4/NKX2-5 protein-protein interactions [38, 39], and it is unknown to what extent these protein-protein interactions have temporal characteristics during the differentiation process, compounds were tested during a broad treatment window (D2–D10) representing both mesodermal commitment and activation of differentiation markers (Fig. 1a). Spontaneous beating is observed on D9 of this assay, in line with activation of venGFP and atrRFP reporters. Both the % of venGFP+ cells and the single cell mean fluorescent intensity (MFI) of venGFP were analyzed for all tested compounds to assess ventricular cardiomyocyte differentiation. To assess differentiation to the atrial fate, atrRFP and the atrRFP/venGFP ratio were also measured for a subset of compounds.

Primary compound screening was conducted for 32 derivatives of GATA4-targeted lead compound 3i-1000 (Supplementary Table S2, Figures S1 and S2), and active compounds were evaluated in follow-up experiments. Statistically significant increases in the %venGFP+ cells were observed for 3i-1000, 3i-1047, 3i-1103, 3i-1148, and 3i-1236 (Fig. 1b). A trend for increases in %venGFP+ cells were observed for 3i-1194 (Fig. 1b), 3i-2042, 3i-2043, and 3i-2045 (Supplementary Figure S1a). All-trans retinoid acid (ATRA), a known inhibitor of embryonic multipotent CPs [47], induced statistically significant dose-dependent decreases in the %venGFP+ cells, as expected (Fig. 1b).

The single-cell mean fluorescent intensity (MFI) was also measured in order to understand the levels of activation of marker gene expression in cardiac cells at a single-cell level (Fig. 1c, Supplementary Figures S1b, S2b). As GATA4-targeted compounds inhibit NKX2-5-dependent transcription [38, 39], and germline deletion of NKX2-5 in mouse embryos resulted in detectable, but downregulated expression of the venGFP promoter sequence (encoded by Myl2) [32], a decline in venGFP MFI at a single cell level would be consistent with previous models of NKX2-5 regulation of ventricular transcriptional networks. Indeed, statistically significant decreases in venGFP-MFI were observed upon compound treatment with 3i-1000, 3i-1103, 3i-1148, and ATRA (Fig. 1c). Additionally, there was a tendency of venGFP-MFI to decrease upon treatment with 3i-1047, 3i-1165 (Fig. 1c), and 3i-2043 (Supplementary Figure S1b). We therefore concluded that active compounds increased the proportion of venGFP+ cells, but venGFP levels in these cells decline compared to control samples, consistent with expected effects of interruption of GATA4/NKX2-5 interactions.

### Subclass of GATA4-targeted compounds possessing an acetyl lysine-like domain associated with ventricular differentiation

**Fig. 2:**
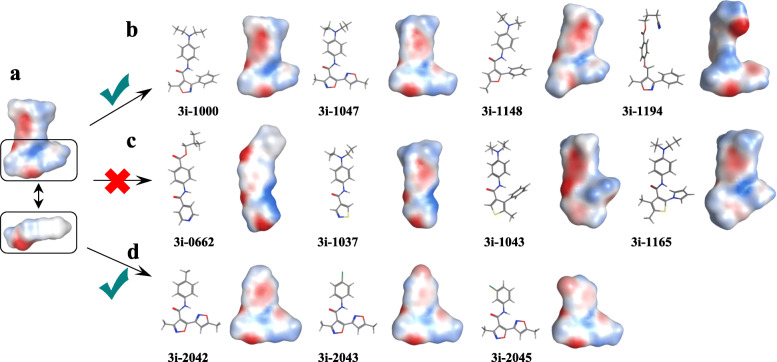
Subclass of structure-dependent GATA4/NKX2-5 synergy inhibitors demonstrate a substantial increase of %venGFP+ cells. **a** Electrostatic distribution of compound 3i-1000 partially resembles the electrostatic field of an acetyl lysine residue. **b** Subclass of GATA4/NKX2-5 synergy inhibitors 3i-1000, 3i-1047, 3i-1148, and 3i-1194 contain the acetyl lysine-like domain and increased the %venGFP+ cells in the spontaneous differentiation assay (*n* = 3–26). **c** Highly potent GATA4/NKX2-5 synergy inhibitors 3i-0662, 3i-1037, 3i-1043, and 3i-1165 are not structurally aligned with the acetyl lysine-like domain and do not activate ventricular gene expression (*n* = 2–3). **d** Low affinity GATA4/NKX2-5 synergy inhibitors 3i-2042, 3i-2043, and 3i-2045 demonstrated a trend to increase the number of venGFP+ cells in the differentiation assay (*n* = 2)

In order to determine the effects of compounds on stem cell differentiation, venGFP was utilized to rank the activity of GATA4-targeted compounds in the spontaneous differentiation assay. Based on our previous studies [38, 39], a group of 32 structurally or functionally similar compounds (Supplementary Table S2) were selected for screening experiments (*n* > = 2). Screening results (Fig. 1b*,* Supplementary Figures S1a, S2a) reveal a consistent finding: all compounds that were either inactive (90–110%, 10 out of 32 compounds) or agonistic (> 110%, 3 out of 32 compounds) in previous GATA4/NKX2-5 synergy studies (Supplementary Table S2) showed no significant change (> twofold) of %venGFP+ cells in spontaneous differentiation experiments. However, a number of the inhibitory compounds in the GATA4/NKX2-5 synergy assay (< 90%, 19 out of 32 compounds) were able to demonstrate an effect on the number of venGFP+ cells. Therefore, screening data of 32 compounds demonstrate that GATA4/NKX2-5 inhibitory activity is preferred for the chemical modulation of ventricular differentiation. A more detailed structural comparison of low energy conformations revealed a structure-activity relationship with an independent subclass of GATA4/NKX2-5 inhibitory compounds that contributed to %venGFP+ cells (Fig. 2). Computational analysis of active compounds indicates a common nominator for the subclass of GATA4/NKX2-5 inhibitory compounds that mimics the electrostatic field of an acetylated lysine residue. Strikingly, the most potent venGFP-activating compounds, e.g., 3i-1000, 3i-1047, 3i-1148, and 3i-1194 (excluding compound 3i-1103 with different chemotype), carry the electrostatically similar acetyl lysine-like domain that separates those from other potent GATA4/NKX2-5 synergy inhibitors, e.g., 3i-0662, 3i-1037, 3i-1043, and 3i-1165. Additionally, another structurally similar set of compounds, namely 3i-2042, 3i-2043, and 3i-2045 (Fig. 2) demonstrated a trend to increase the number of venGFP+ cells.

### Modulation of atrial gene expression programs by GATA4-targeted compounds

**Fig. 3:**
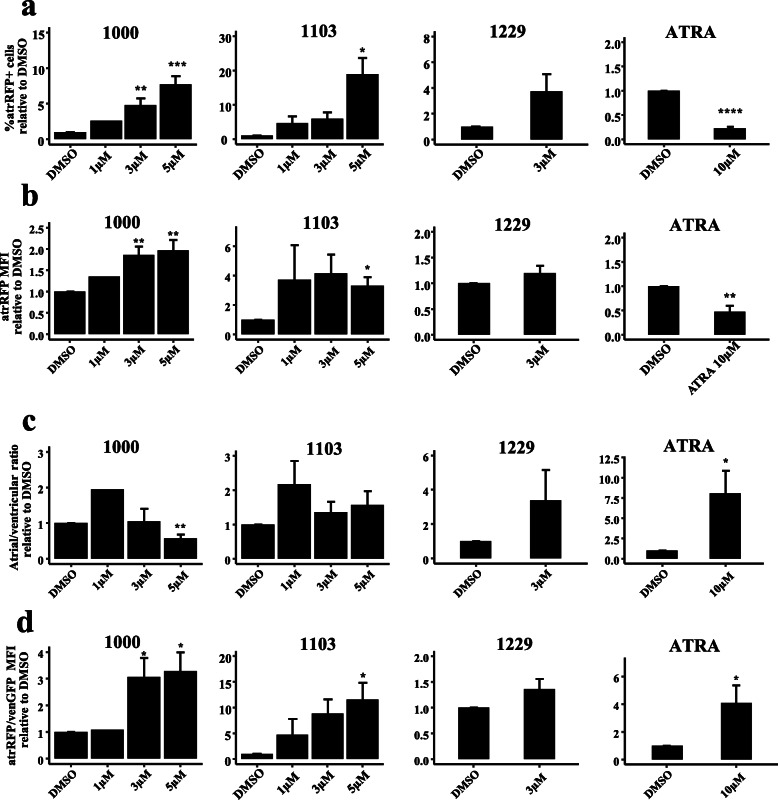
Compound screening for activation of atrial and ventricular reporter genes. GATA4-targeted compounds were screened during D2–D10 of spontaneous differentiation and measured for **a** %atrRFP+ cells, **b** atrRFP-MFI, **c** atrial/ventricular ratio, and **d** atrRFP/venGFP ratio. *All-trans* retinoic acid (ATRA), atrRFP (SMyHC3-TdTomato, atrial), and mean fluorescent intensity (MFI). Data is presented as mean ± SEM (*n* = independent experiments). ****P ≤ 0.0001, ***P ≤ 0.001, **P ≤ 0.01, *P ≤ 0.05 (*T* test vs DMSO control). *n* = 2 (3i-1000 1 μM), *n* = 3 (3i-1229 3 μM), *n* = 4 (3i-1103 1 μM, 3i-1103 3 μM, 3i-1103 5 μM), *n* = 9 (3i-1000 3 μM, 3i-1000 5 μM), *n* = 10 (ATRA 10 μM)

In addition to the analysis of ventricular reporter expression, expression of an atrial reporter was examined for a subset of compounds. Importantly, in addition to being the earliest atrial-specific marker during embryogenesis, the atrRFP promoter was shown to be positively regulated by GATA4, but not by GATA4 co-activators NKX2-5 or MEF2C [48]. The expression of atrial markers was also affected by treatment with novel compounds, reflected by changes in the %atrRFP cells and the atrRFP-MFI (Fig. 3a, b*,* Supplementary Figures S3a-b). Statistically significant increases in %atrRFP+ cells were observed following treatment with 3i-1000 and 3i-1103 (Fig. 3a). An increasing trend in the %atrRFP cells were observed following treatment with 3i-1228 (Supplementary Figure S3a) and 3i-1229 (Fig. 3a), whereas a statistically significant decrease in the %atrRFP+ cells was only observed upon treatment with ATRA (Fig. 3a). Statistically significant increases in atrRFP-MFI were observed upon treatment with 3i-1000, 3i-1103 (Fig. 3b), 3i-1235, 3i-1236, and 3i-1238 (Supplementary Figures S3b). Thus, the potent GATA4-targeted compounds 3i-1000 and 3i-1103 were also potent activators of atrRFP expression.

The effects of atrialization of CPs were observed by analysis of %atrRFP/%venGFP and atrRFP/venGFP-MFI (Fig. 3c, d*,* Supplementary Figure S4a-b). Statistically significant decreases in the atrial/ventricular ratio were observed for 3i-1000 (Fig. 3c, 5 μM), suggesting that this compound promotes the differentiation of ventricular, rather than atrial cardiomyocytes. Only ATRA led to statistically significant increases in atrial/ventricular ratio, consistent with previous reports [43, 49], though trends for increases were observed for 3i-1000 (1 μM), 3i-1103 (1 μM), and 3i-1229 (3 μM) (Fig. 3c). Statistically significant increases in atrRFP/venGFP-MFI were observed for 3i-1000, 3i-1103, ATRA (Fig. 3d), and 3i-1238 (Supplementary Figure S4b). Intriguingly, these results demonstrate the differential modulation of both atrial and ventricular gene expression by GATA4-targeted compounds during the differentiation of PSCs to the cardiomyocyte fate.

### Alteration of expression of cell identity genes during pluripotent stem cell differentiation

**Fig. 4:**
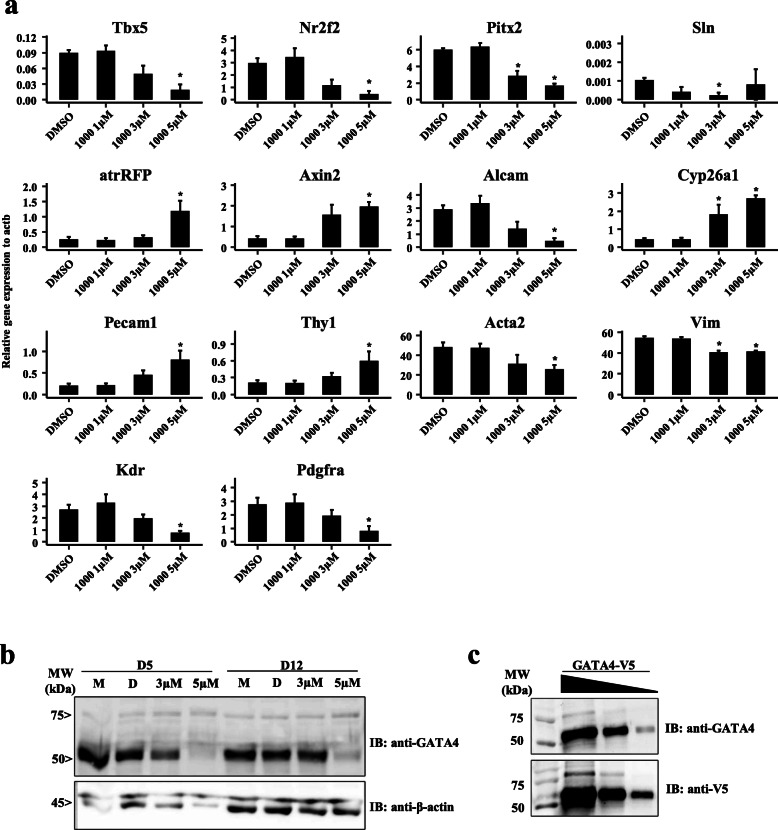
Characterization of chemically induced differentiation by qRT-PCR and immunoblotting. **a** D12 cultures were collected and analyzed by qRT-PCR for markers of cell identity genes, transcription factors, and signaling pathways. Data is presented as mean ± SEM (*n* = 4, independent experiments). *P ≤ 0.05 (Wilcoxon test vs DMSO control). **b** Modulation of GATA4 isoforms by compound treatment in differentiating mESCs. Differentiating mESCs were treated with 3i-1000 during D2–D10 window of differentiation and collected for immunoblotting at D5 and D12. Anti-GATA4 detection, lanes 1–4 (day 5 samples); lane 1: medium only (M), lane 2: DMSO (D), lane 3: 3i-1000 (3 μM), lane 4: 3i-1000 (5 μM), and lanes 5–8 (D12 samples) lane 5: medium only (M), lane 6: DMSO (D), lane 7: 3i-1000 (3 μM), lane 8: 3i-1000 (5 μM). Note the heavier GATA4-70 kDa band is abundant in differentiating mESCs. **c** For confirmation of this band as GATA4, overexpression of GATA4-V5 in HEK293 cells and immunoblotting for anti-GATA4 and anti-V5. Lane 1: protein ladder, lane 2: GATA4-V5, lane 3: GATA4-V5 (1/5), and lane 4: GATA4-V5 (1/25). Note that GATA4-70 kDa is detected in cells with overexpression by both anti-GATA4 and anti-V5 antibodies

In order to gain insight into the full scope of changes in gene expression induced by the GATA4-targeted compounds, qRT-PCR was performed on D12 EBs after ten-day treatment with the lead compound 3i-1000 (1 μM, 3 μM, 5 μM) for markers of cardiomyocytes (pan-, atrial-, ventricular), CPs, stromal cells, cardiac transcription factors, and developmental signaling pathways previously implicated in cardiomyocyte differentiation and cardiac subtype specification. In-depth characterization was restricted to 3i-1000 based on the beneficial effects of this compound demonstrated in previous studies [38–40, 50], in addition to its possession of an acetyl lysine-like fragment characteristic of compounds promoting ventricular gene expression. The results are shown in Fig. 4a and Supplementary Figure S5. Statistically significant declines were observed for cardiac transcription factors Tbx5, Nr2f2 and Pitx2 (Fig. 4a), suggesting perturbation of the cardiac gene regulatory network by 3i-1000. Though the atrial-specific Sln showed a statistically significant decline at 3 μM, no changes where observed at the concentration of 5 μM 3i-1000. However, atrRFP showed a statistically significant increase when treated with 5 μM 3i-1000 (Fig. 4a), suggesting this may be a key threshold for atrial-specification. Furthermore, non-canonical Wnt marker Alcam showed a statistically significant decrease, whereas canonical Wnt signaling marker Axin2 was upregulated. Statistically significant upregulation of the retinoic acid degrading gene Cyp26a1 was also observed (Fig. 4a). Furthermore, endothelial marker Pecam1 and fibroblast marker Thy1 were upregulated by 3i-1000 treatment, whereas the smooth muscle marker Acta2 and fibroblast marker Vim displayed statistically significant declines. Finally, progenitor markers Kdr and Pdgfra were downregulated in EBs, suggesting that a more differentiated phenotype is induced by 3i-1000 treatment (Fig. 4a), Importantly, Gata4 mRNA levels were unchanged by 3i-1000 (Supplementary Figure S5).

### GATA4 protein levels during the differentiation of chamber-specific mESCs

To further elucidate the mechanism-of-action of the lead compound 3i-1000 and its regulation of atrial and ventricular gene expression, we measured GATA4 protein levels during the spontaneous differentiation of mESCs. Similar to primary screening experiments, EBs were cultured in the presence of 3i-1000 during D2–D10 of differentiation, and EBs were collected for immunoblotting at D5 and D12 (Fig. 4b and Supplementary Figure S6). Treatment with 3i-1000 decreased GATA4 protein levels (50 kDa band), and this effect was more pronounced with addition of a higher concentration of 3i-1000 (Fig. 4b). Surprisingly, we also observed a heavier protein band with an estimated molecular weight of 70 kDa (GATA4-70 kDa) (Fig. 4b). To confirm that the 70 kDa band maintained by 3i-1000 was not the result of non-specific binding of the GATA4 antibody, GATA4 with a V5-tag was overexpressed in HEK293 cells (Fig. 4c and Supplementary Figure S7). Indeed, the V5-antibody recognized both 50 kDa and 70 kDa bands in samples with GATA4-V5 overexpression, but not in samples without overexpression of GATA4-V5, indicating that the GATA4-70 kDa band observed in mESC differentiation experiments indeed represents GATA4 protein.

### Stage-specific addition of GATA4-targeted compounds promotes the differentiation of multipotent CPs to ventricular cardiomyocytes

**Fig. 5:**
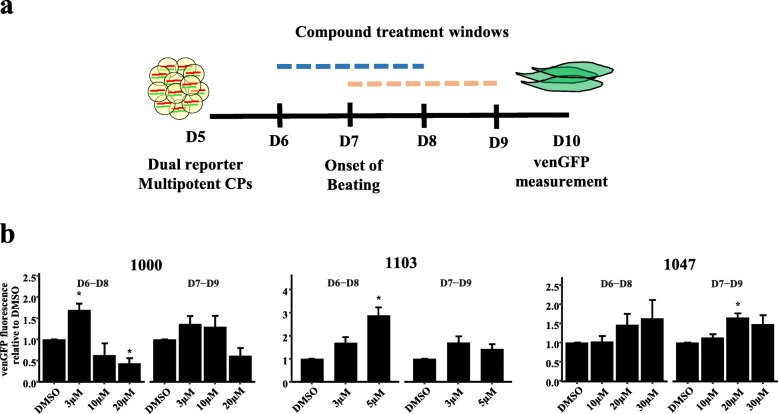
**a** Strategy for confirmatory assay for the cardiogenic activity of novel compounds. Compounds were added either prior to (D6–D8, cardiac progenitors) or after (D7–D9, cardiomyocytes) the onset of spontaneous beating in defined, serum-free conditions. **b** venGFP total fluorescence upon compound treatment with 3i-1000, 3i-1103, and 3i-1047. Cardiac progenitor (CP) and venGFP (Myl2-eGFP, ventricular). Data is presented as mean ± SEM (*n* = 4, independent experiments). *P < 0.05 (*T* test vs DMSO control)

In order to more precisely define effects of GATA4-targeted compounds on multipotent cardiac progenitors, we next explored whether shorter compound treatment windows might promote cardiomyocyte differentiation in a defined cardiac progenitor cell assay. For this purpose, a second assay was utilized based on a directed differentiation system with defined progenitor cell populations (Fig. 5a) [43]. Importantly, D6 cells represent second heart field CPs expressing Isl1, as described previously [43], and beating is never observed prior to D7 in this assay, assuring their undifferentiated phenotype. In contrast to the primary screening assay, which was based on flow cytometry, this assay is based on total venGFP fluorescence. Though total fluorescence was used as measurement in the compound screening assay, we observed cardiomyocyte differentiation efficiencies of ~ 10–40% in basal conditions as measured by flow cytometry in directed differentiation. Compounds were tested during two windows, one in which compounds were administered before the onset of spontaneous beating (D6–D8), and one in which compounds were administered after the onset of spontaneous beating (D7–D9). Selected compounds tested in this assay showed previous activity within the primary spontaneous differentiation assay (Fig. 5b, Supplementary Figure S8). Both 3i-1000 (3 μM) and 3i-1103 (5 μM) induced statistically significant increases in venGFP total fluorescence when added to CPs, but not to differentiated cardiomyocytes (Fig. 5b). Addition of cytotoxic (20 μM) concentrations of 3i-1000 led to a statistically significant decline in venGFP signal, as expected. Furthermore, 3i-1047 (20 μM) induced statistically significant increases in venGFP total fluorescence when added after the onset of spontaneous beating. Thus, GATA4-targeted compounds 3i-1000 and 3i-1103 also promote ventricular cardiomyocyte differentiation programs in multipotent CPs.

### Analysis of the protein interactome by BioID

**Fig. 6:**
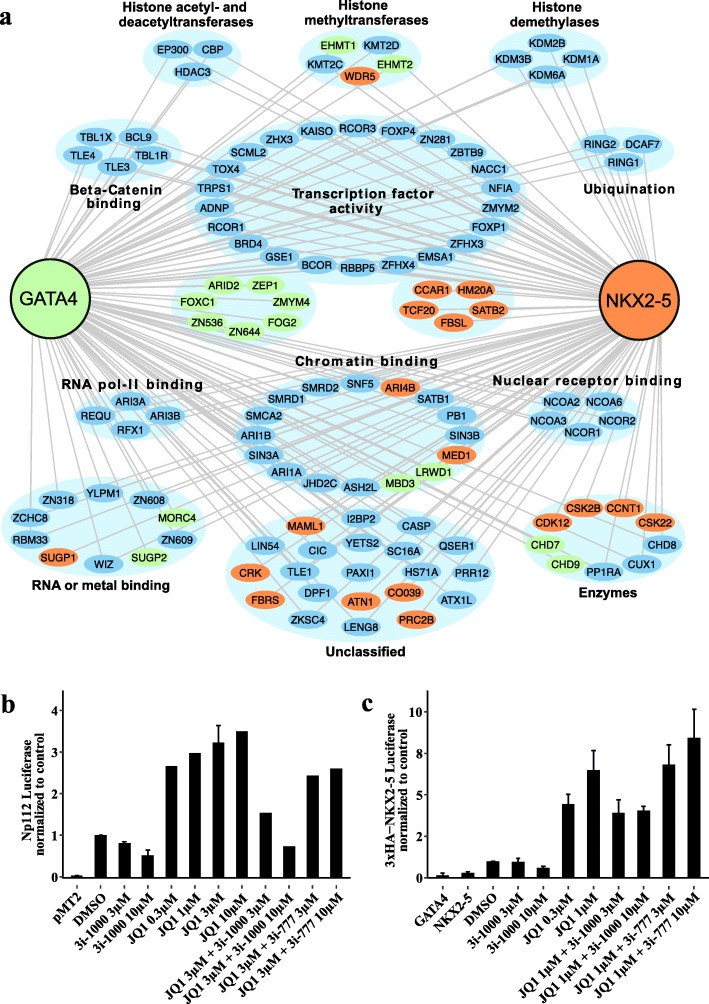
**a** Protein networks of GATA4 and NKX2-5 in HEK293 cells identified by single BioID experiment reveal overlapping functional protein classes regulating transcriptional pathways. Proteins detected by BioID were filtered using the CRAPome contaminant repository. Proteins seen in more than 10% (41/411) of CRAPome database experiments were discarded. Then, the top 100 interactors with the highest peptide-spectrum match (PSM) values were illustrated for both GATA4 and NKX2-5. **b** Regulation of the expression of a chamber-specific reporter reveals joint transcriptional modulation by GATA4/NKX2-5 and BET bromodomain inhibitors. Modulation of transcriptional activity resulting from GATA4 binding sites and GATA4 overexpression. NP112 (Nppb promoter sequence) was transfected into COS-1 cells in combination with a GATA4 overexpression vector and luciferase activity was measured in the presence or absence of GATA4-targeted compounds and/or the BET bromodomain inhibitor (+)-JQ1. **c** Modulation of transcriptional activity resulting from activation of a 3xHA-NKX2-5 luciferase cassette containing NKX2-5 binding sites in combination with GATA4/NKX2-5 overexpression in the presence or absence of GATA4-targeted compounds and/or the BET bromodomain inhibitor (+)-JQ1. NP112—rat minimal BNP promoter-luciferase construct containing GATA4 binding sites, pMT2—plasmid backbone only (no TF overexpression), 3xHA-NKX2-5—promoter-luciferase construct containing three high affinity NKX2-5 binding sites upstream of a rat albumin minimal promoter. Data is presented as mean ± SEM (*n* ≥ 2, independent experiments)

In order to gain further insight into GATA4 interactions that could underlie 3i-1000 mechanisms-of-action, particularly in regard to novel GATA4 binding partners affecting atrial and ventricular differentiation, we analyzed the protein interactome of GATA4 and NKX2-5 by BioID (Fig. 6a*,* Supplementary Figure S9 and Tables S3, S4). During this experiment, GATA4 or NKX2-5 was expressed in HEK293 cells and biotinylated proteins were identified by mass spectrometry. Gene ontology enrichment analysis of BioID results demonstrated a comparable protein-protein interactome for both TFs GATA4 and NKX2-5 (Supplementary Tables S3 and S4)*.* TFs and their co-factors, chromatin and RNA polymerase II binders, and regulators of epigenetic signaling are highly enriched among the most abundant co-proteins. BioID results also demonstrate the frequent interaction/proximity of GATA4 with bromodomain-containing protein 4 (BRD4).

Importantly, bromodomain-containing proteins interact with acetylated lysines on histones [51], overlapping with the acetyl lysine-like domain within the ventricular differentiation inducers of the subclass of GATA4-targeted compounds (see the “Subclass of GATA4-targeted compounds possessing an acetyl lysine-like domain associated with ventricular differentiation” section). To further explore the role of the acetyl lysine-like domain associated with cardiogenic compounds, we conducted comparisons of sequence conservation in zinc fingers of GATA4 among different species (Supplementary Figure S10). It is well-recognized that human and other mammals are unable to regenerate cardiomyocytes after birth or injury [52] and that they also carry a conserved arginine (R310) at the C-terminal tail of the zinc finger domain. To our great surprise, regenerative species such as zebrafish, Eastern newt, bat star, sea cucumber, African clawed frog, and *Hydra vulgaris* carry conserved expression of lysine (K310) at the same site.

### Combinatorial effects of GATA4-targeted compounds and BET bromodomain inhibitor on activation of chamber-specific reporter gene expression

Finally, in order to rule out the possibility that GATA4-targeted compounds interact directly with bromodomains via their acetyl lysine-like fragment, compounds 3i-1000 and 3i-1047 were screened in the BromoMAX assay (Supplementary Figure S11). These experiments revealed that compounds 3i-1000 and 3i-1047 were inactive against bromodomain proteins and, therefore, did not have affinity across those protein target classes. However, intrigued by the possibility that chemical modulation of epigenetic signaling might regulate GATA4-dependent transcription, we tested the BET bromodomain inhibitor (+)-JQ1 [53] in reporter assays using either a GATA-dependent promoter (NP112) or an NKX2-5 dependent promoter (3xHA-NKX2-5), respectively. Importantly, NP112 promoter sequences are originally from the chamber-specific Nppb gene, and NKX2-5 is known as a master regulator of ventricular cell fate determination and maturation [54]. Overexpression of either GATA4 alone or the combination of GATA4 and NKX2-5 was performed in conjunction with compound treatment. Strikingly, (+)-JQ1 increased GATA4-mediated transcription from the NP112 promoter, and this was attenuated by treatment with 3i-1000 (Fig. 6b). Similarly, (+)-JQ1 treatment led to increases in reporter activity from the NKX2-5-dependent 3xHA-NKX2-5 promoter in conjunction with GATA4/NKX2-5 overexpression, and this was also attenuated by 3i-1000 (Fig. 6c). However, exposure to other bromodomain inhibitors PFI-3 (probable global transcription activator SNF2L2 (SMARCA2) and transcription activator BRG1 (SMARCA4)) and GSK4027 (histone acetyltransferase KAT2A and histone acetyltransferase KAT2B) did not change luciferase-activity in GATA4/NKX2-5 reporter assays (data not shown). Thus, selective BET family bromodomain inhibition leads to modulation of GATA4 activity, and this is further modulated by the GATA4-targeted lead compound 3i-1000. The GATA/NKX synergy activator, 3i-0777 (Supplementary Table S2), was not able to inhibit (+)-JQ1-mediated increases in GATA-dependent transcription.

### Analysis of global transcriptional changes in differentiated primary ventricular cardiomyocytes

**Fig. 7:**
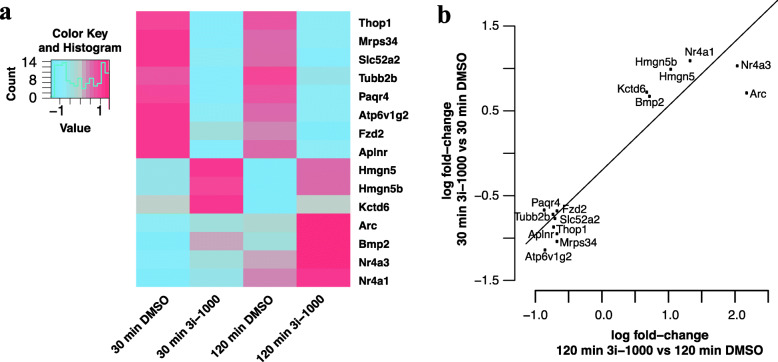
GRO-seq experiment (two biological replicates pooled for GRO-seq analysis). **a** Heat map depicting differentially expressed genes following 30 and 120 min treatments with GATA4-targeted compound 3i-1000 versus DMSO control in neonatal rat ventricular cardiomyocytes. **b** Most prominently up- and downregulated genes following GRO-seq experiments

Intrigued by the possibility that GATA4-targeted compounds could also modulate the maturation of fully differentiated ventricular cardiomyocytes, we measured the effect of compound 3i-1000 on transcription in primary neonatal rat ventricular myocytes by global run-on and sequencing (GRO-seq) to identify pathways affected by 3i-1000 treatment (Fig. 7a, b and Supplementary Table S5). Identification and quantification of gene regions that changed their transcriptional activity was performed after short-term exposure of 3i-1000 (30 and 120 min) to focus on direct effects on transcriptional regulation, rather than indirect effects resulting from prolonged compound treatment. The results revealed an upregulation of early response genes, e.g., activity-regulated cytoskeleton-associated protein (Arc), and orphan nuclear receptor proteins (NR4A-family) (Fig. 7a, b)*.* GRO-seq results also indicated chemical modulation of cardiac cell fate regulators in ventricular cardiomyocytes, such as bone morphogenetic protein, which was upregulated following two-hour exposure to 3i-1000*.*

## Discussion

Transplantation of PSC-derived cardiac progenitors [2] and cardiomyocytes [8] is being actively explored as a therapeutic modality to replace tissue lost following myocardial infarction, but it is uncertain if transplanted cells are able to maintain a fully differentiated phenotype in the adult heart. Furthermore, it is unclear whether these cells are able to assume subtype-specific gene expression programs characteristic of mature atrial and ventricular cardiomyocytes [8]. Small molecule compounds might represent a viable strategy to overcome these challenges, though the in vivo utility of compounds that induce cardiomyocyte differentiation by targeting ubiquitous signaling pathways might be limited due to unwanted systemic effects. Furthermore, these compounds do not direct differentiation to specific subtype fates, such as atrial and ventricular cardiomyocytes [22–26]. The development of small molecule compounds that could enhance the therapeutic potential of exogenous delivery of stem cell-derived cardiac progenitors [2], cardiomyocytes [8], or cardiospheres [10] to the infarcted heart would represent a significant advance in the field of cardiac regeneration.

**Fig. 8:**
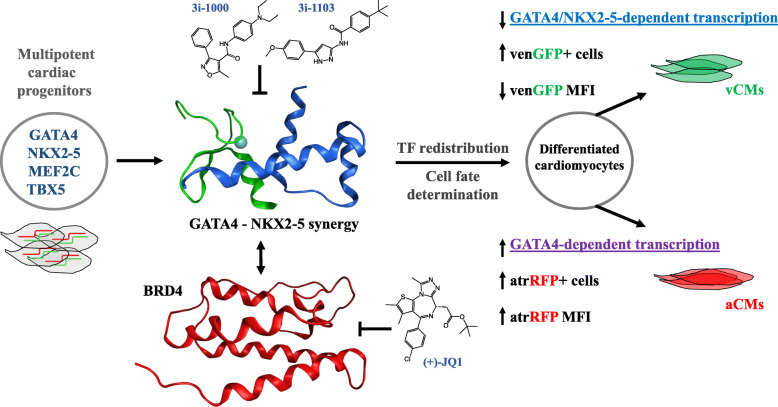
Summary model figure depicting proposed actions of GATA4-targeted compounds and the bromodomain inhibitor (+)-JQ1 on cardiac gene regulatory networks. Ventricular cardiomyocytes (vCMs), atrial cardiomyocytes (aCMs), bromodomain-containing protein 4 (BRD4), transcription factor (TF), atrRFP (SMyHC3-TdTomato, atrial), and venGFP (Myl2-eGFP, ventricular)

In the present study, we explored the effects of GATA4-targeted compounds on the expression of atrial and ventricular reporter genes in differentiating PSCs and identified a structural subclass that distinctly alters atrial and ventricular gene expression. These compounds target the GATA4/NKX2-5 interaction, a tissue-specific combination that might confer selectivity towards the heart [8]. Treatment of differentiating PSCs with 3i-1000 and 3i-1103, previously identified inhibitors of the GATA4/NKX2-5 protein-protein interaction [38, 39], resulted in an increased proportion of venGFP+ cells, while leading to decreases in venGFP-MFI measured at the single cell level. This observation is in line with reported phenotypes arising from germline deletion of NKX2-5 [32, 55]. In addition to altering expression of ventricular reporters, GATA4-targeted compounds induced an increase in both atrRFP+ cells and atrRFP-MFI. As atrRFP is known to be GATA4, but not NKX2-5 dependent [48], this increase in GATA4-dependent transcription could be explained by TF repositioning in response to interruption of TF synergy, as has been described previously in genetic loss-of-function models [27]. This suggests that novel GATA4-targeted compounds specifically affect TF interactions and that inhibition of these interactions might enhance the activity of a single TF at some promoter sequences. Our approach thus includes the comprehensive modulation of TF machinery, as inhibition of the GATA4/NKX2-5 interaction allows for more efficient cardiac gene activation/differentiation via alternative synergistic/repressive GATA4 complexes. Therefore, we have created a model in which GATA4-targeted compounds modulate atrial and ventricular target gene expression during the differentiation process (Fig. 8).

Compounds detailed within the present study target the GATA4/NKX2-5 protein-protein interaction, and protein-protein interactions have only recently been considered as a new type of drug target for small molecules [56]. Protein–protein interactions regulate a variety of cellular functions, including cell cycle progression, signal transduction, and metabolic pathways [57]. However, targeting protein-protein interactions with a small molecule compound is a challenging task, and protein-protein interactions have previously been considered as “undruggable,” although the paradigm is now changing due to more and more successful examples [58]. Protein-protein networks are highly interconnected, and challenges also lie in the development of reliable primary assays and in the identification of valid positive hits. Furthermore, identification of the target site and structure-based design of new compounds may be challenging if the proteins of interest have not yet been co- crystallized. Indeed, the precise mechanism-of-action involving the impact on TF interactions must be further resolved in order to develop more selective compounds with in vitro and/or in vivo bioactivity.

In the present study, structural analysis of GATA4/NKX2-5 inhibitory compounds highlights a scaffold that carries a position-specific molecular fragment mimicking the electrostatics of an acetyl lysine domain. Furthermore, sequence-based comparison revealed a GATA4-specific point-mutation (R310K) that associates with regenerative species and may represent a promising target site for compound design and gene editing. The association of the arginine-lysine switch with the function of GATA4 in regenerative species is currently unclear, and evaluation of common genetic variation among human populations (dbSNP Build 153) did not recognize GATA4-mutation R310K (data not shown). Therefore, K310 conserved in regenerative species may represent a modification that will have an impact on compound design and could be introduced by genome editing in a therapeutic setting. Furthermore, computational modeling suggests direct binding of GATA-targeted compounds to the C-terminal zinc finger of GATA4 that mediates DNA binding and the majority of protein-protein interactions (unpublished observations). Interestingly, BioID analysis of the GATA4 interactome revealed interactions between GATA4 and bromodomain-containing proteins, such as BRD4. We further showed novel functional crosstalk between GATA4 and BRD4 by using the BET bromodomain inhibitor (+)-JQ1 and reporter gene assays derived from the chamber-specific Nppb promoter. Notably, GATA4-mediated transcription was regulated independently by either GATA4- and/or BRD4-acting compounds. These results suggest an important role for the GATA4-BRD4-axis in the regulation of chamber-specific gene expression and could indicate a framework through which novel therapeutic avenues could be developed. Interestingly, BET bromodomain inhibition has previously been shown to be beneficial in animal model of heart failure [59].

In addition to GATA4/NKX2-5 synergy inhibition, compound binding will most likely have a broad impact on the cardiac TF network. Indeed, characterization of cells resulting from chemically induced differentiation with GATA-targeted compounds revealed that long-term inhibition of GATA4/NKX2-5 affected the mRNA levels of several cardiac TFs, cell markers, and differentiation pathways measured by qRT-PCR. A potential limitation of this analysis is that bulk qRT-PCR is not necessarily suitable for the analysis of heterogenous populations, such as embryoid bodies. Another limitation was that we used predefined non-toxic concentrations for several compounds (e.g., 3i-1040, 3i-1212, 3i-1234 and 3i-1238) that may have accordingly limited their ability to engage their target protein and induce cell differentiation and thus influenced the interpretation of the results. However, by utilizing a shorter compound treatment window (48 h) in a defined directed differentiation assay, 3i-1000 and 3i-1103 increased venGFP expression in multipotent CPs, but exerted no effects on reporter expression when added after the onset of spontaneous beating. This suggests that the GATA4/NKX2-5 interaction might be necessary for CP maintenance and self-renewal and that this can be perturbed chemically to induce differentiation. Of note, we have previously characterized a structurally distinct subclass of GATA4-acting compounds that causes stem cell toxicity, and during these differentiation studies, there were no compounds able to overcome those structural preconditions [60]. Decreased GATA4-protein levels have been shown to associate with stem cell death [61, 62], and here decreased GATA4 protein levels (50 kDa band) were observed particularly at the higher concentration of 3i-1000. Additionally, we observed declines in GATA4 protein levels but not declines in Gata4 mRNA levels upon 3i-1000 treatment in differentiating PSCs. We and others have previously observed that GATA4 mRNA levels remain constant despite declines in protein levels due to extensive post-transcriptional and post-translational regulation of GATA4 during both homeostasis and in certain disease models [63, 64].

In addition to the alteration of gene expression in differentiating stem cells, GATA-targeted compounds were shown to modulate gene expression in differentiated, primary ventricular cardiomyocytes, including the upregulation of Arc, NR4-related proteins, and bone morphogenetic proteins (BMPs). Arc protein has been previously reported to have a cardioprotective role by inhibiting apoptosis and preserving mitochondrial integrity [65]. The NR4A-family protein Nurr77 (NR4A2) has been linked to muscle regeneration and cardiac remodeling, as well as to cardioprotection and regulation of cardiac apoptosis [66]. BMP-2, a secreted protein necessary for both mesodermal formation and cardiogenesis, was upregulated after 2-h exposure to 3i-1000 [67]. Interestingly, BMP-2 also regulates patterning of the atrioventricular canal and the regulation of both atrial and ventricular identity [68]. Thus, GRO-seq in ventricular cardiomyocytes provided additional evidence that 3i-1000 regulates cardiomyocyte subtype gene programs.

## Conclusion

In conclusion, our findings indicate that pharmacological targeting of cardiac TFs with a structural subclass of GATA-targeted compounds allows selective alteration of atrial and ventricular differentiation in PSCs. Though the TF networks for influencing cardiac progenitor differentiation towards the cardiac fate are well-recognized, the molecular mechanisms of chemically induced effects on TFs and epigenetics remains poorly understood. In order to achieve further progress in the field, novel physical, genetic, and pharmacological interventions are needed to uncover previously unrecognized molecular level mechanisms of cardiac differentiation. Here, we show that GATA4-dependent transcription remains at the core of progenitor cell signaling which leads to cardiac differentiation. Additionally, these experiments resulted in the identification of compounds selectively regulating atrial and ventricular gene expression, as well as provided insight into the mechanism-of-action of novel GATA4-targeted compounds involving an acetyl lysine-like subdomain. Moreover, the data presented here could lead to further refinement of GATA-targeted compounds, with the hope of developing targeted therapies for the treatment of heart diseases. Indeed, the compound scaffolds uncovered in the present study could be used to develop cardiac regenerative strategies based on the pharmacological modulation of cell fate determination of exogenously delivered or endogenous cardiac progenitor cells to specific atrial and ventricular subtypes.

## Supplementary Information

**Additional file 1: Supplementary Figure S1.** Primary screening of GATA4-targeted compounds in differentiating stem cells for the activation of a ventricular reporter gene (venGFP, Myl2-eGFP). Compounds were screened during D2-D10 window of spontaneous differentiation of mouse embryonic stem cells (mESCs). Differentiation cultures were measured on D12 of differentiation after treatment with GATA4-targeted compounds for (**a)** %Myl2-eGFP (ventricular, venGFP+) cells out of total cell population, (**b)** the mean fluorescent intensity (MFI) of venGFP. Data is presented as mean (*n* ≥ 2, independent experiments). **Supplementary Figure S2.** Compound screening for activation of ventricular reporter gene (venGFP, Myl2-eGFP). Compounds were screened during D2-D10 window of spontaneous differentiation of mouse embryonic stem cells (mESCs). Differentiation cultures were measured on D12 of differentiation after treatment with GATA4-targeted compounds for (**a)** %Myl2-eGFP (ventricular, venGFP+) cells out of total cell population, (**b)** the mean fluorescent intensity (MFI) of venGFP. Data is presented as mean ± SEM (*n* ≥ 3, independent experiments). **Supplementary Figure S3.** Compound screening for activation of atrial reporter gene (SMyHC3-TdTomato, atrRFP). Compounds were screened during D2-D10 window of spontaneous differentiation of mouse embryonic stem cells (mESCs). Differentiation cultures were measured on D12 of differentiation after treatment with GATA4-targeted compounds and measured for (**a)** %SMyHC3-TdTomato (atrial, atrRFP+) cells out of total cell population, (**b)** the mean fluorescent intensity (MFI) of atrRFP. Data is presented as mean ± SEM (*n* ≥ 3 (1228, *n* = 2), independent experiments). **P < 0.01, *P < 0.05 (T-test vs DMSO control). **Supplementary Figure S4.** Ratio of expression between ventricular and atrial reporter genes. Compounds were screened during D2-D10 window of spontaneous differentiation of mouse embryonic stem cells (mESCs). Differentiation cultures were measured on D12 of differentiation after treatment with GATA4-targeted compounds and measured for **(a)** atrial/ventricular ratio and (**b)** atrRFP/venGFP ratio. Data is presented as mean ± SEM (*n* ≥ 3 (1228, *n* = 2), independent experiments). **P < 0.01, *P < 0.05 (T-test vs DMSO control). **Supplementary Figure S5.** Characterization of chemically induced differentiation by qRTPCR. D12 cultures were collected and analyzed for markers of cell identity genes, transcription factors, progenitors, and signaling pathways. Data is presented as mean ± SEM (*n* = 4, independent experiments). *P < 0.05 (Wilcoxon test vs DMSO control). **Supplementary Figure S6.** Original whole Western blot images. Differentiating mouse embryonic stem cells (mESCs) were treated with compound 3i-1000 during D2-D10 window of differentiation and collected at D5 and D12. The cells were lysed into 1% SDS in 50 mM Tris-HCl and protein concentration was determined. **a** On first experiment 80 μg of protein was loaded on gel and **b** on second experiment 30 μg protein was loaded on gel. Membranes were immunoblotted (IB) at first with anti-GATA4 antibody and after strip wash with anti-β-actin antibody. Samples: medium only (M), DMSO (D), 3i-1000 (3 μM), 3i-1000 (5 μM). Independent experiments were repeated two times. **Supplementary Figure S7.** Original whole Western blot images for HEK-cells with GATA4-V5 Tet-On/Off overexpression. The cells were lysed into 4′ Laemmli buffer with 2-mercaptoethanol. From the crude cell lysate, a sample was diluted 1/5 and further 1/25 with 1′ Laemmli buffer. A 10 μl sample from each dilution was loaded on gel and immunoblotted (IB) with GATA4 or V5 antibodies. **a** For control, HEK-cells were transfected with rtTA, the samples were prepared similarly as for GATA4-V5 overexpression and loaded on gel with decreasing amount 1/1, 1/5, 1/25. **b** At the second repetition, for control, the cells with GATA4-V5 overexpression were lysed into RIPA-buffer, protein concentration was determined and 5, 10 and 16 μg samples were loaded on gel. Independent experiments were repeated two times. **Supplementary Figure S8.** GATA4-targeted compounds promote differentiation of ventricular cardiomyocytes in a directed differentiation assay. Compounds were added either prior to (D6-D8, cardiac progenitors) or after (D7-D9, cardiomyocytes) the onset of spontaneous beating in defined, serum-free conditions. Total fluorescence of ventricular reporter gene (venGFP, Myl2-eGFP) upon compound treatment is depicted for compounds 3i-1148, 3i-1120, 3i-1165, and 3i-1194. Data is presented as mean ± SEM (*n* = 4, independent experiments). **Supplementary Figure S9.** Protein interactome of GATA4 and NKX2-5 by BioID after CRAPomefiltering. Proteins in more than 10% (41/411) of CRAPome database experiments were discarded. **Supplementary Figure S10*.*** High sequence conservation in zinc finger domain of GATA4 among different species. Human and other mammals are unable to regenerate cardiomyocytes after birth and express an arginine (hR310, purple) at the C-terminal tail of the zinc finger. However, species with regenerative capacity, including Zebrafish (Q09JY7), Eastern newt (F2W888), Bat star (Q6XZF5), Sea cucumber (A0A2G8JQ98), African clawed frog (Q91677) and Hydra vulgaris (T2MH05) have consistent expression of lysine at the same position (green). Conserved residues responsible for C4-coordination of zinc fingers are highlighted with gray color. In the bottom row, the alignment results are represented as follows: The asterisk (*) indicates a single and fully conserved residue. A colon (:) indicates conservation between groups of strongly similar properties. A period (.) indicates conservation between groups with weakly similar properties. A number at the end of the line indicates the running number of the last amino acid of the respective sequence. Protein sequences were downloaded from UniProt Knowledgebase (UniProtKB) which contains two separate sections; UniProtKB/Swiss-Prot (SP, manually annotated) and UniProtKB/TrEMBL (TR, computationally annotated). Sequences were aligned by using Clustal Omega (European Bioinformatics Institute, EMBL-EBI). **Supplementary Figure S11.** BromoMAX assay shows no significant perturbation of bromodomain proteins in cell-free assays by compounds 3i-1000 and 3i-1047 at 10 μM, indicating that the acetyl-lysine like domain within the compounds do not bind directly to bromodomains. **Supplementary Table S1*.*** Taqman assays used for the characterization of embryoid bodies from chemically induced differentiation experiments. **Supplementary Table S2.** Structural derivatives of GATA-targeted compounds [3, 5] examined for stage-specific activation of atrial and ventricular reporter genes in differentiating pluripotent stem cells. **Supplementary Table S3.** Gene ontology (GO) enrichment analysis of CRAPome-filtered BioIDresults for GATA4 (320 identified proteins) determines the most abundant functional associations. **Supplementary Table S4.** Gene ontology (GO) enrichment analysis of CRAPome-filtered BioIDresults for NKX2-5 (359 identified proteins) determines the most abundant functional associations. **Supplementary Table S5.** Summary of differentially expressed genes following 30 and 120 min treatments with GATA4-targeted compound 3i-1000 versus DMSO control in neonatal rat ventricular cardiomyocytes.

## Data Availability

All data generated or analyzed during this study are included in this published article and its supplementary information files.

## Abbreviations

ATRA: *All-trans* retinoic acid
atrRFP: SMyHC3-TdTomato
BET: Bromodomain and extraterminal domain
BMP: Bone morphogenetic proteins
CP: Cardiac progenitor
EB: Embryoid body
ESC: Embryonic stem cell
GRO-seq: Global run-on sequencing
mESC: Mouse embryonic stem cell
MFI: Mean fluorescent intensity
NRVM: Neonatal rat ventricular myocytes
PSC: Pluripotent stem cell
TF: Transcription factor
venGFP: Myl2-eGFP
