# Supplementary material for: GATA-targeted compounds modulate cardiac subtype cell differentiation in dual reporter stem cell line
Source: Stem Cell Res Ther. 2021 Mar 18;12:190. doi: 10.1186/s13287-021-02259-z (PMC7977156; doi:10.1186/s13287-021-02259-z)
Supplement: Supplementary file 1 — Additional file 1: Supplementary Figure S1. Primary screening of GATA4-targeted compounds in differentiating stem cells for the activation of a ventricular reporter gene (venGFP, Myl2-eGFP). Compounds were screened during D2-D10 window of spontaneous differentiation of mouse embryonic stem cells (mESCs). Differentiation cultures were measured on D12 of differentiation after treatment with GATA4-targeted compounds for (a) %Myl2-eGFP (ventricular, venGFP+) cells out of total cell population, (b) the mean fluorescent intensity (MFI) of venGFP. Data is presented as mean (n ≥ 2, independent experiments). Supplementary Figure S2. Compound screening for activation of ventricular reporter gene (venGFP, Myl2-eGFP). Compounds were screened during D2-D10 window of spontaneous differentiation of mouse embryonic stem cells (mESCs). Differentiation cultures were measured on D12 of differentiation after treatment with GATA4-targeted compounds for (a) %Myl2-eGFP (ventricular, venGFP+) cells out of total cell population, (b) the mean fluorescent intensity (MFI) of venGFP. Data is presented as mean ± SEM (n ≥ 3, independent experiments). Supplementary Figure S3. Compound screening for activation of atrial reporter gene (SMyHC3-TdTomato, atrRFP). Compounds were screened during D2-D10 window of spontaneous differentiation of mouse embryonic stem cells (mESCs). Differentiation cultures were measured on D12 of differentiation after treatment with GATA4-targeted compounds and measured for (a) %SMyHC3-TdTomato (atrial, atrRFP+) cells out of total cell population, (b) the mean fluorescent intensity (MFI) of atrRFP. Data is presented as mean ± SEM (n ≥ 3 (1228, n = 2), independent experiments). **P < 0.01, *P < 0.05 (T-test vs DMSO control). Supplementary Figure S4. Ratio of expression between ventricular and atrial reporter genes. Compounds were screened during D2-D10 window of spontaneous differentiation of mouse embryonic stem cells (mESCs). Differentiation cultures were measured on D12 o [file 13287_2021_2259_MOESM1_ESM.pdf]

Supplementary information

GATA-targeted compounds modulate cardiac subtype cell differentiation in dual reporter stem cell line

Mika J. Välimäki[1+], Robert S. Leigh[1+], Sini M. Kinnunen[1], Alexander R. March[1], Ana Hernández de Sande[2], Matias Kinnunen[3,4], Markku Varjosalo[3,4], Merja Heinäniemi[2], Bogac L. Kaynak[1*], Heikki Ruskoaho[1*]

[1] *Drug Research Program, Division of Pharmacology and Pharmacotherapy, University of Helsinki, Helsinki, Finland*
[2] *Institute of Biomedicine, School of Medicine, University of Eastern Finland, Kuopio, Finland*
[3] *Institute of Biotechnology, University of Helsinki, Helsinki, Finland.*
[4] *Helsinki Institute of Life Science, University of Helsinki, Helsinki, Finland.*
[+]authors contributed equally to this work

[*]Corresponding authors:

Bogac L. Kaynak, Division of Pharmacology and Pharmacotherapy, Faculty of Pharmacy, University of Helsinki, P.O. Box 56, FI-00014 Helsinki, FINLAND
Heikki Ruskoaho, Division of Pharmacology and Pharmacotherapy, Faculty of Pharmacy, University of Helsinki, P.O. Box 56, FI-00014 Helsinki, FINLAND

email:
bogac.kaynak@helsinki.fi
heikki.ruskoaho@helsinki.fi

Contents:
Supplementary Figures S1–S11, pages 2–13
Supplementary Tables S1–S5, pages 14–21
Supplementary Methods, pages 22–24

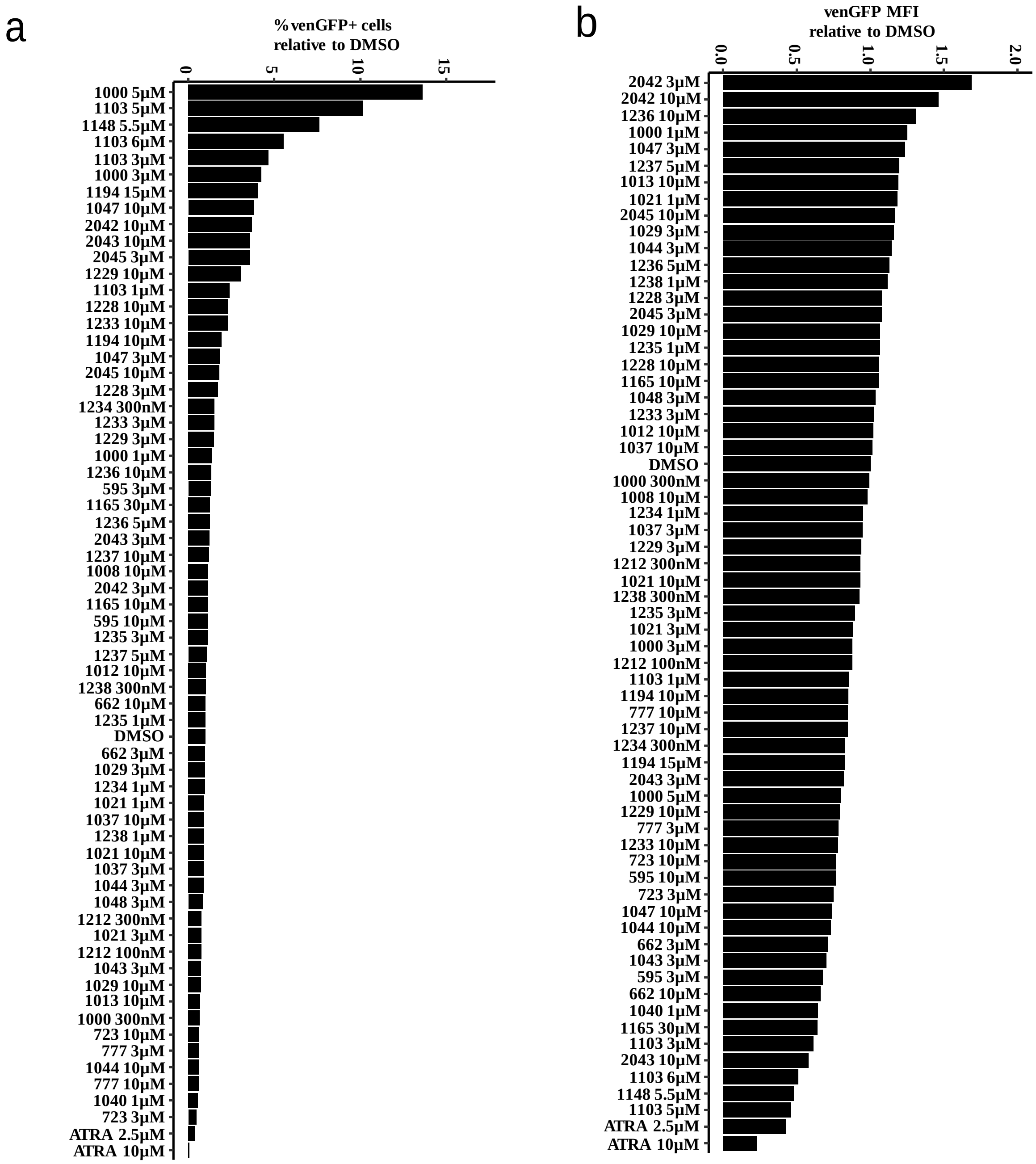

Supplementary Figure S1. Primary screening of GATA4-targeted compounds in differentiating stem cells for the activation of a ventricular reporter gene (venGFP, Myl2-eGFP). Compounds were screened during D2-D10 window of spontaneous differentiation of mouse embryonic stem cells (mESCs). Differentiation cultures were measured on D12 of differentiation after treatment with GATA4-targeted compounds for (**a)** %Myl2-eGFP (ventricular, venGFP+) cells out of total cell population, (**b)** the mean fluorescent intensity (MFI) of venGFP. Data is presented as mean (n ≥ 2, independent experiments).

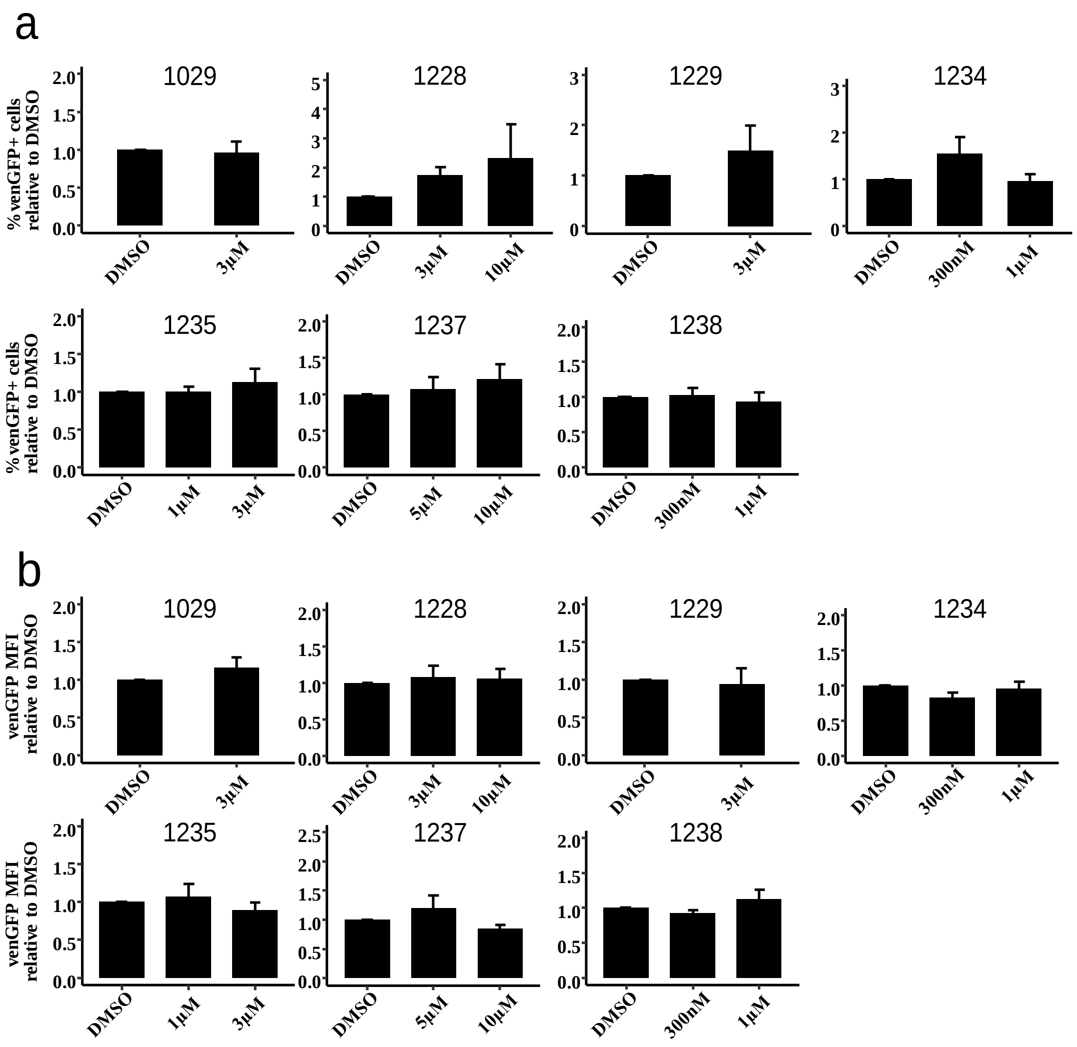

Supplementary figure S2. Compound screening for activation of ventricular reporter gene (venGFP, Myl2-eGFP). Compounds were screened during D2-D10 window of spontaneous differentiation of mouse embryonic stem cells (mESCs). Differentiation cultures were measured on D12 of differentiation after treatment with GATA4-targeted compounds for (**a**) %Myl2-eGFP (ventricular, venGFP+) cells out of total cell population, (**b**) the mean fluorescent intensity (MFI) of venGFP. Data is presented as mean ± SEM (n ≥ 3, independent experiments).

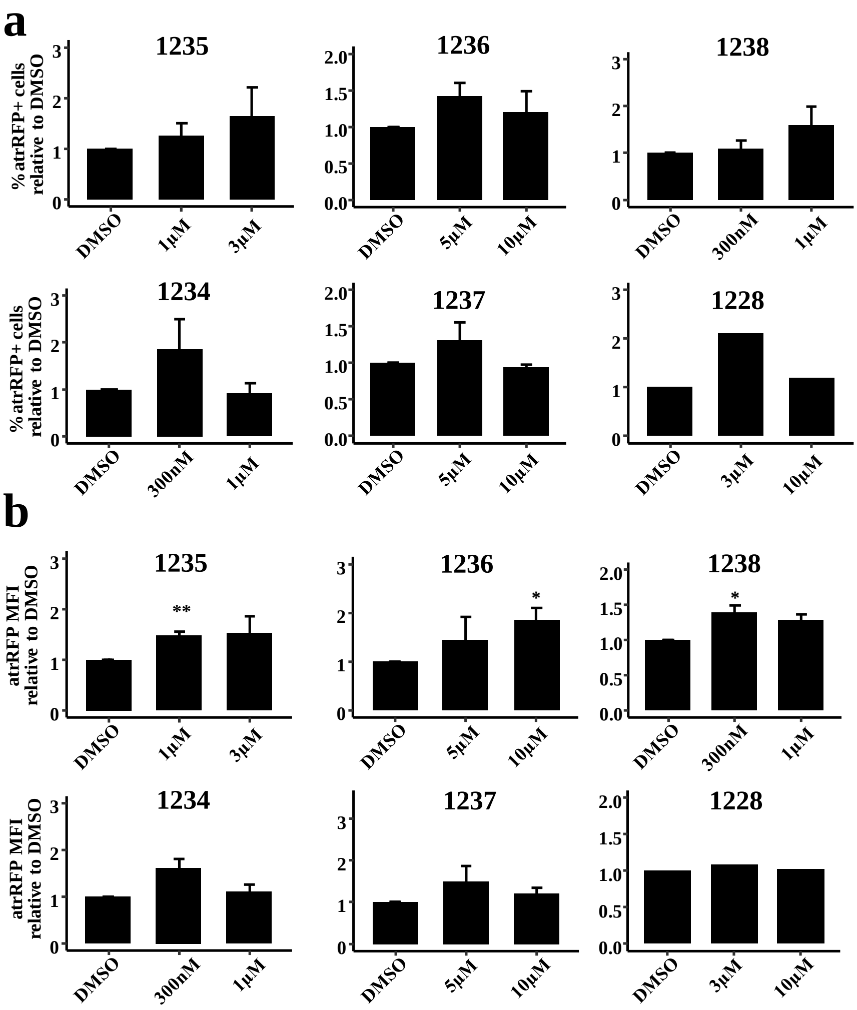

Supplementary Figure S3. Compound screening for activation of atrial reporter gene (SMyHC3-TdTomato, atrRFP). Compounds were screened during D2-D10 window of spontaneous differentiation of mouse embryonic stem cells (mESCs). Differentiation cultures were measured on D12 of differentiation after treatment with GATA4-targeted compounds and measured for (**a)** %SMyHC3-TdTomato (atrial, atrRFP+) cells out of total cell population, (**b)** the mean fluorescent intensity (MFI) of atrRFP. Data is presented as mean ± SEM (n ≥ 3 (1228, n=2), independent experiments). **P < 0.01, *P < 0.05 (T-test vs DMSO control).

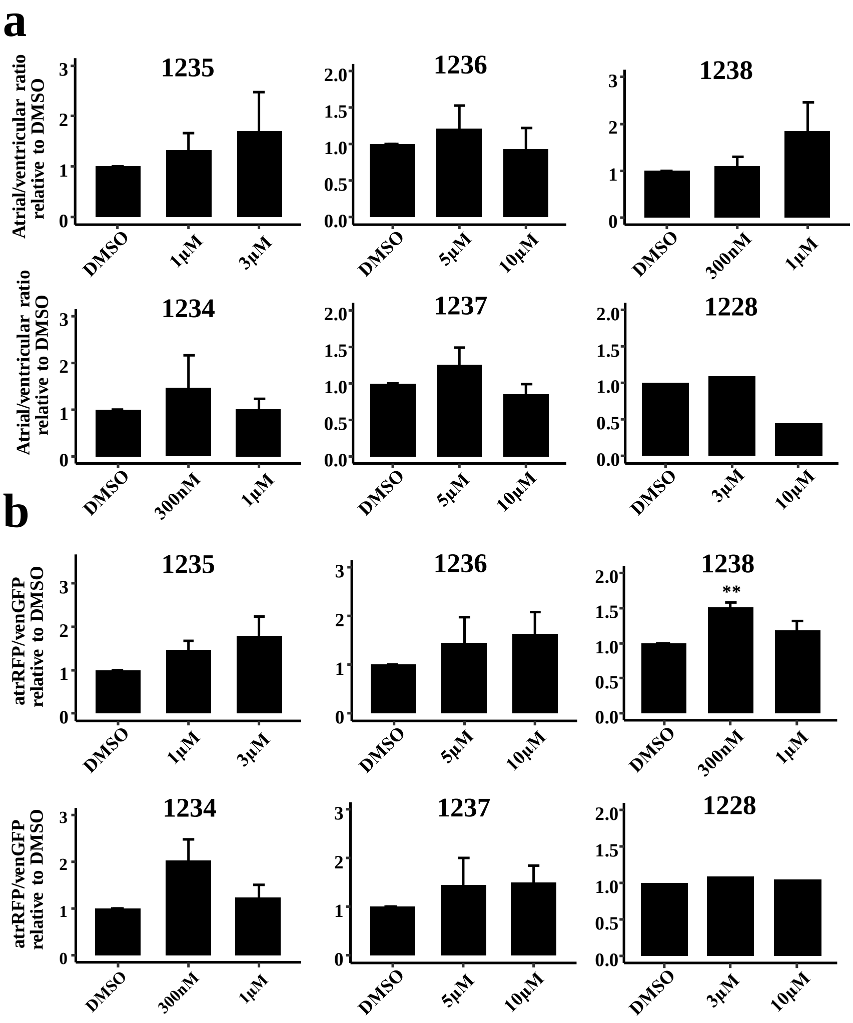

Supplementary Figure S4. Ratio of expression between ventricular and atrial reporter genes. Compounds were screened during D2-D10 window of spontaneous differentiation of mouse embryonic stem cells (mESCs). Differentiation cultures were measured on D12 of differentiation after treatment with GATA4-targeted compounds and measured for **(a)** atrial/ventricular ratio and (**b)** atrRFP/venGFP ratio. Data is presented as mean ± SEM (n ≥ 3 (1228, n=2), independent experiments). **P < 0.01, *P < 0.05 (T-test vs DMSO control).

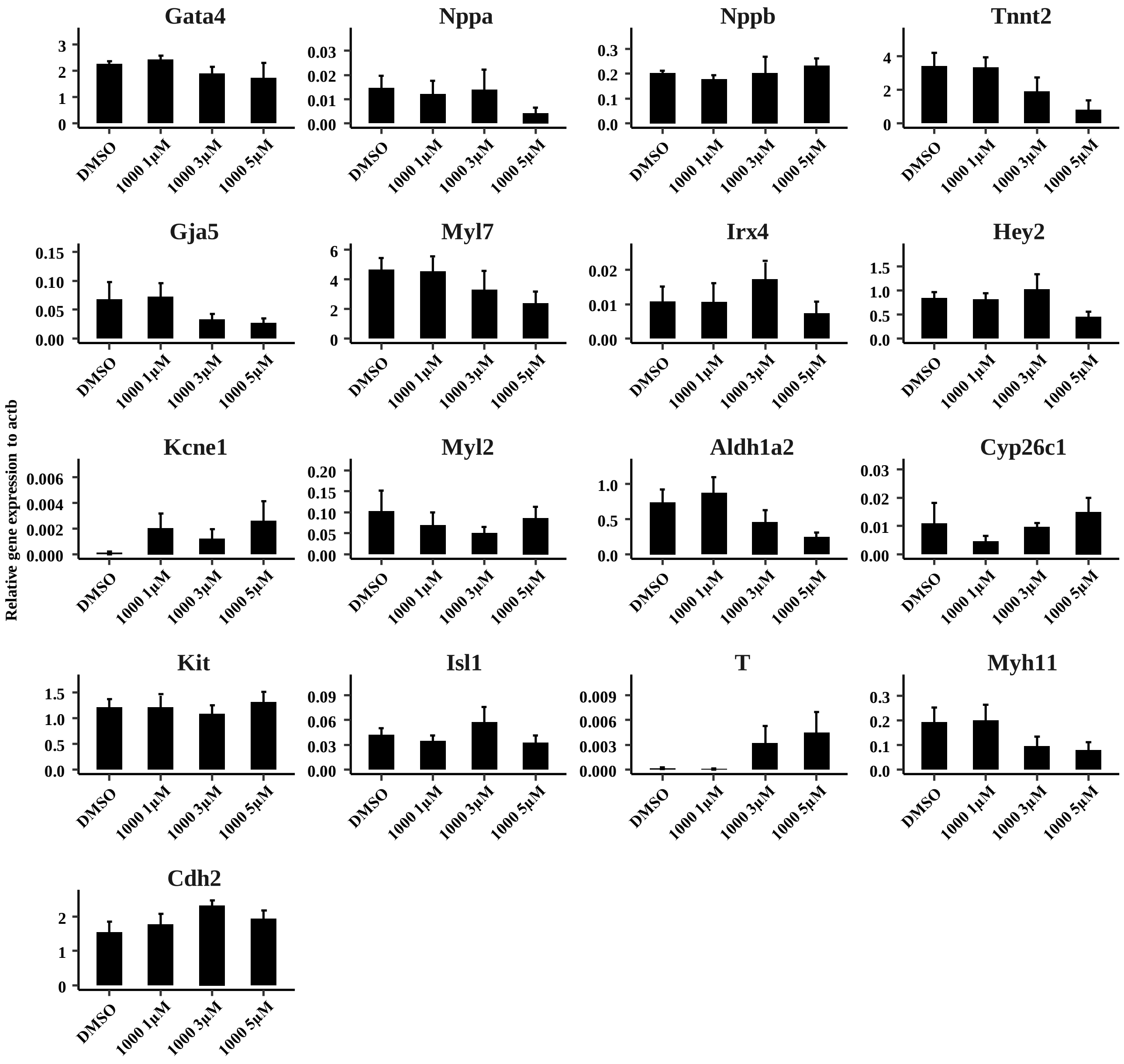

Supplementary Figure S5. Characterization of chemically-induced differentiation by qRT-PCR. D12 cultures were collected and analysed for markers of cell identity genes, transcription factors, progenitors, and signalling pathways. Data is presented as mean ± SEM (n=4, independent experiments). *P < 0.05 (Wilcoxon test vs DMSO control).

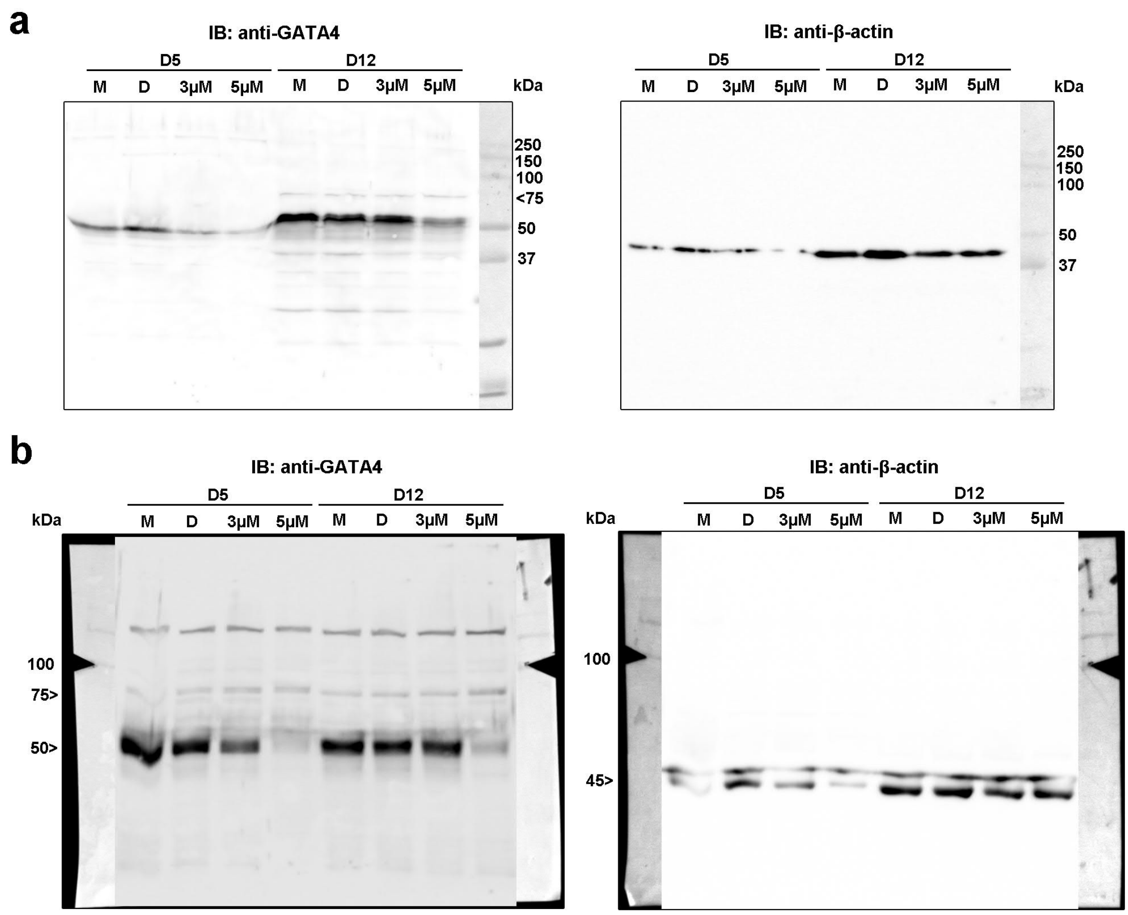

Supplementary Figure S6. Original whole Western blot images. Differentiating mouse embryonic stem cells (mESCs) were treated with compound 3i-1000 during D2-D10 window of differentiation and collected at D5 and D12. The cells were lysed into 1% SDS in 50mM Tris-HCl and protein concentration was determined. **a** On first experiment 80 µg of protein was loaded on gel and **b** on second experiment 30 µg protein was loaded on gel. Membranes were immunoblotted (IB) at first with anti-GATA4 antibody and after strip wash with anti-β-actin antibody. Samples: medium only (M), DMSO (D), 3i-1000 (3 µM), 3i-1000 (5 µM). Independent experiments were repeated two times.

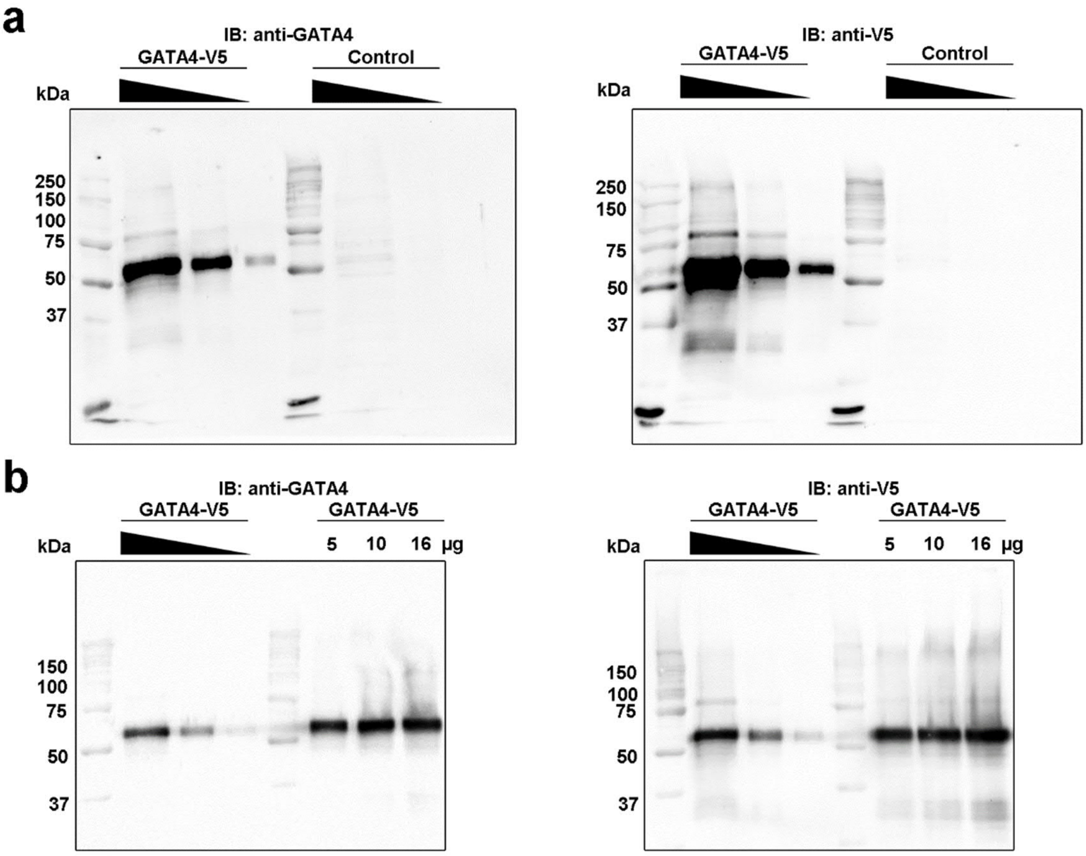

Supplementary Figure S7. Original whole Western blot images for HEK-cells with GATA4-V5 Tet-On/Off overexpression. The cells were lysed into 4× Laemmli buffer with 2-mercaptoethanol. From the crude cell lysate, a sample was diluted 1/5 and further 1/25 with 1× Laemmli buffer. A 10 µl sample from each dilution was loaded on gel and immunoblotted (IB) with GATA4 or V5 antibodies. **a** For control, HEK-cells were transfected with rtTA, the samples were prepared similarly as for GATA4-V5 overexpression and loaded on gel with decreasing amount 1/1, 1/5, 1/25. **b** At the second repetition, for control, the cells with GATA4-V5 overexpression were lysed into RIPA-buffer, protein concentration was determined and 5, 10 and 16 µg samples were loaded on gel. Independent experiments were repeated two times.

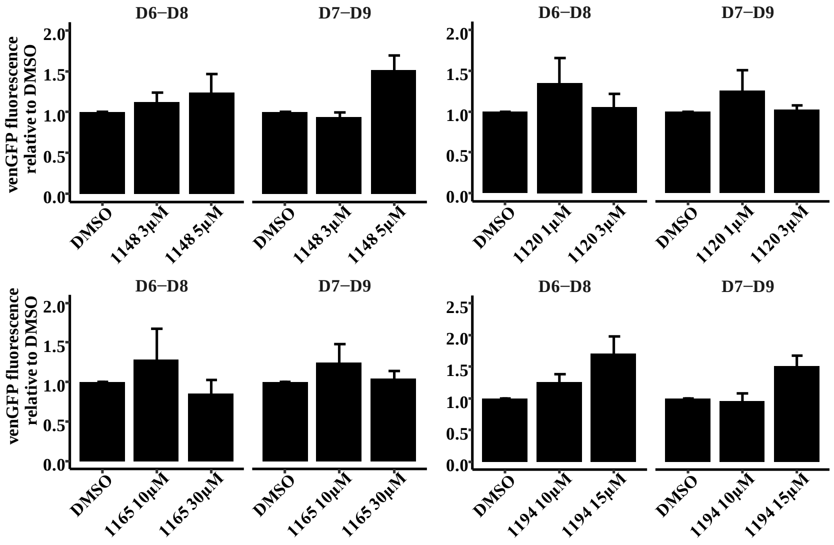

Supplementary Figure S8. GATA4-targeted compounds promote differentiation of ventricular cardiomyocytes in a directed differentiation assay. Compounds were added either prior to (D6-D8, cardiac progenitors) or after (D7-D9, cardiomyocytes) the onset of spontaneous beating in defined, serum-free conditions. Total fluorescence of ventricular reporter gene (venGFP, Myl2-eGFP) upon compound treatment is depicted for compounds 3i-1148, 3i-1120, 3i-1165, and 3i-1194. Data is presented as mean ± SEM (n = 4, independent experiments).

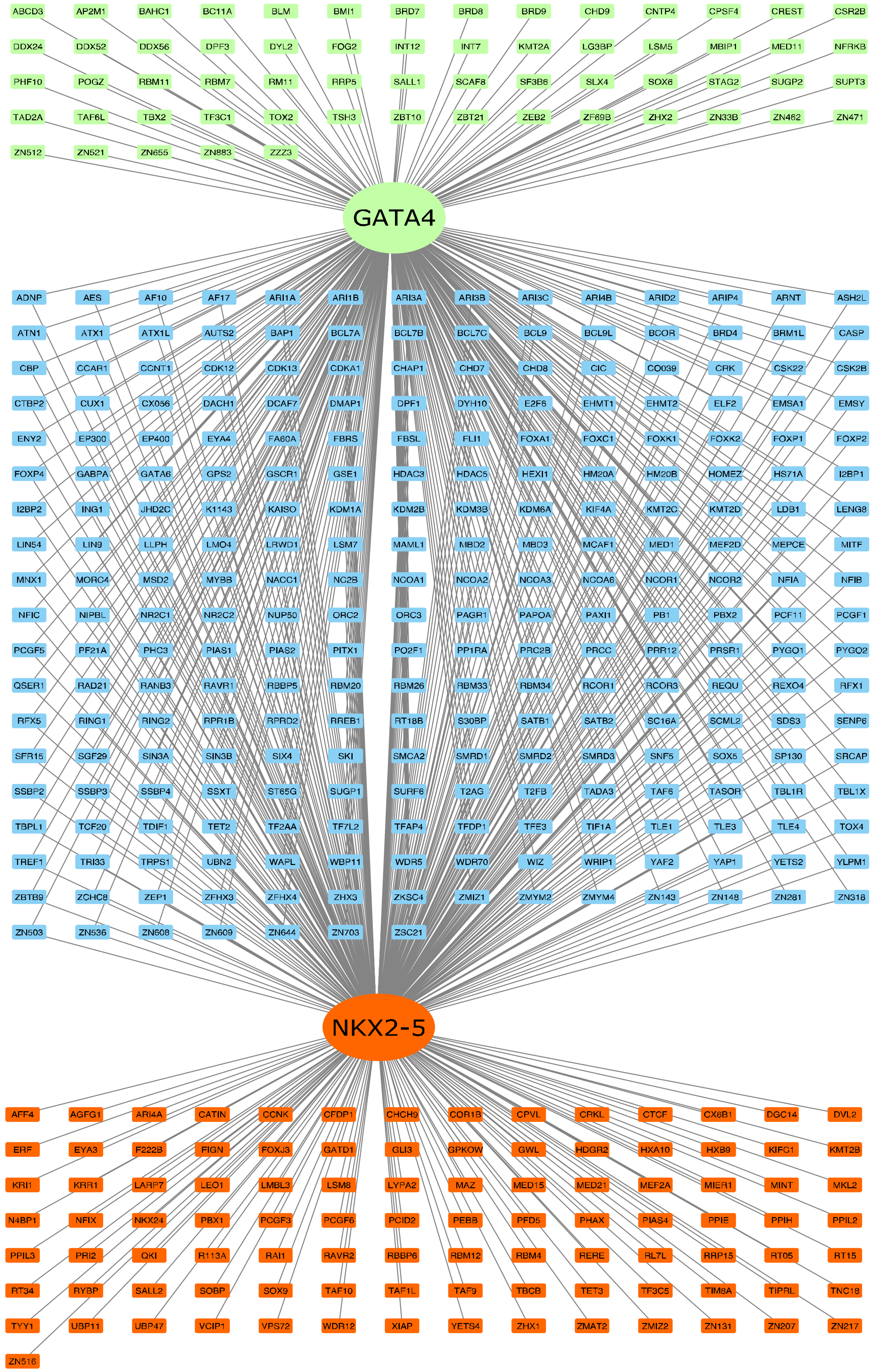

Supplementary Figure S9. Protein interactome of GATA4 and NKX2-5 by BioID after CRAPome-filtering. Proteins in more than 10% (41/411) of CRAPome database experiments were discarded.

```
TR|Q09JY7|Zebrafish         GAMSTPLWRRDGTGHYLCNACGLYHKMNGINRPLVKPQRRL-SASRRVGLSCTNCQTTTT 232
TR|F2W888|Eastern newt      ------------------------YHKMNGINRPLIKPQRRL-SASRRVGLSCANCHTTTIT 36
TR|Q6XZF5|Bat star          GAISTPLWRRDGTGHYLCNACGLYHKMNGMSRPLIKPQRRLQSGSRREGIVCANCHTTTT 423
TR|A0A2G8JQ98|Sea cucumber  GAISTPLWRRDGTGHYLCNACGLYHKMNGYNRPLLKPQRRM-SGSRREGITCANCHTSTT 92
SP|Q91677|Afr. clawed frog  GAMSTPLWRRDGTGHYLCNACGLYHKMNGINRPLIKPQRRL-SASRRVGLSCANCHTTTT 246
TR|T2MH05|Hydra vulgaris    GATSTPLWRRDTRGHYLCNACGLYHKMNGANRPLIKPKRRL-SQARRTGIVCSNCKTSQT 294
SP|Q08369|Mouse             GAMSTPLWRRDGTGHYLCNACGLYHKMNGINRPLIKPQRRL-SASRRVGLSCANCQTTTT 278
SP|P43694|Human             GAMSTPLWRRDGTGHYLCNACGLYHKMNGINRPLIKPQRRL-SASRRVGLSCANCQTTTT 279
SP|P46152|Rat               GAMSTPLWRRDGTGHYLCNACGLYHKMNGINRPLIKPQRRL-SASRRVGLSCANCQTTTT 278
SP|Q0Q0E4|Dog               GAMSTPLWRRDGTGHYLCNACGLYHKMNGINRPLIKPQRRL-SASRRVGLSCANCQTTTT 279
TR|I3LUZ9|Pig               GAMSTPLWRRDGTGHYLCNACGLYHKMNGINRPLIKPQRRL-SASRRVGLSCANCQTTTT 279
SP|P43691|Chicken           GAMSTPLWRRDGTGHYLCNACGLYHKMNGINRPLFKPQRRL-SASRRVGLSCANCHTTTT 219
TR|A0A2I3SFW5|Chimpanzee    GAMSTPLWRRDGTGHYLCNACGLYHKMNGINRPLIKPQRRL-SASRRVGLSCANCQTTTT 280
TR|M3WLT5|Cat               GAMSTPLWRRDGTGHYLCNACGLYHKMNGINRPLIKPQRRL-SASRRVGLSCANCQTTTT 279
TR|F1N551|Bovine            GAMSTPLWRRDGTGHYLCNACGLYHKMNGINRPLIKPQRRL-SASRRVGLSCANCQTTTT 279
TR|F6QQ97|Rhesus macaque    GAMSTPLWRRDGTGHYLCNACGLYHKMNGINRPLIKPQRRL-SASRRVGLSCANCQTTTT 250
TR|H9G5S5|Am. chameleon     GAMSTPLWRRDGTGHYLCNACGLYHKMNGINRPLIKPQRRL-SASRRIGLMCANCQTTTT 253
TR|F7CJW8|Horse             GAMSTPLWRRDGTGHYLCNACGLYHKMNGINRPLIKPQRRL-SASRRVGLSCANCQTTTT 279
TR|F7B924|Opossum           GAMSTPLWRRDGTGHYLCNACGLYHKMNGINRPLIKPQRRL-SASRRVGLSCANCQTTTT 290
TR|H2PPJ2|Sum. orangutan    GAMSTPLWRRDGTGHYLCNACGLYHKMNGINRPLIKPQRRL-SASRRVGLSCANCQTTTT 279
TR|G1SH13|Rabit             GAMSTPLWRRDGTGHYLCNACGLYHKMNGINRPLIKPQRRL-SASRRVGLSCANCQTTTT 280
TR|M3XRU8|Ferret            GAMSTPLWRRDGTGHYLCNACGLYHKMNGINRPLIKPQRRL-SASRRVGLSCANCQTTTT 279
TR|A0A096N2A9|Olive baboon  GAMSTPLWRRDGTGHYLCNACGLYHKMNGINRPLIKPQRRL-SASRRVGLSCANCQTTTT 279
TR|A0A0D9RWJ0|Green monkey  GAMSTPLWRRDGTGHYLCNACGLYHKMNGINRPLIKPQRRL-SASRRVGLSCANCQTTTT 279
TR|G1LHY0|Giant panda       GAMSTPLWRRDGTGHYLCNACGLYHKMNGINRPLIKPQRRL-SASRRVGLSCANCQTTTT 285
TR|A0A3Q0CU09|Golden hamster GAMSTPLWRRDGTGHYLCNACGLYHKMNGINRPLIKPQRRL-SASRRVGLSCANCQTTTT 277
TR|H0ZSU3|Zebra finch       GAMSTPLWRRDGTGHYLCNACGLYHKMNGINRPLFKPQRRL-SASRRVGLSCANCHTTTT 219
TR|A0A452E0B6|Goat          GAMSTPLWRRDGTGHYLCNACGLYHKMNGINRPLIKPQRRL-SASRRVGLSCANCQTTTT 279
TR|W5Q0U8|Sheep             GAMSTPLWRRDGTGHYLCNACGLYHKMNGQNRPLIKPKRRL-SASRRVGLSCANCQTTTT 183
TR|A0A2K5V779|Cyn. monkey   GAMSTPLWRRDGTGHYLCNACGLYHKMNGINRPLIKPQRRL-SASRRVGLSCANCQTTTT 262
TR|G3SQY9|African elephant  GAMSTPLWRRDGTGHYLCNACGLYHKMNGINRPLIKPQRRL-SASRRVGLSCANCQTTTT 280
                                                    ****** .***.**:**: * :** *: *:**:*: *

TR|Q09JY7|Zebrafish         TLWRRNAEGEPVCNACGLYMKLHGVPRPLAMKKEGIQTRKRKPKNISKTKPGSSEGQS-- 290
TR|F2W888|Eastern newt      TLWRRNAEGEPVCNACGLYMKLHGVPRPLAMKKEGIQTRKRKPKNLNKSNTSTGSTSS-E 95
TR|Q6XZF5|Bat star          TLWRRNKEGEPVCNACGLYYKLHSVNRPLAMKKDGIQTRKRKPKGSSKQQQQQVNGQQ-Q 482
TR|A0A2G8JQ98|Sea cucumber  TLWRRNKEGEPVCNACGLYYKLHSVNRPLAMKKDGIQTRKRKPKKQTQQQQTPTSQSA-N 151
SP|Q91677|Afr. clawed frog  TLWRRNAEGEPVCNACGLYMKLHGVPRPLAMKKEGIQTRKRKPKNLSKSKTLTGQSGS-D 305
TR|T2MH05|Hydra vulgaris    TLWRRNGSGEPVCNACGLYYKLHKVNRPLTMKKDGIQTRNRKSTGKNKKLKKESSIMN-E 353
SP|Q08369|Mouse             TLWRRNAEGEPVCNACGLYMKLHGVPRPLAMRKEGIQTRKRKPKNLNKSKTPAGPAG--E 336
SP|P43694|Human             TLWRRNAEGEPVCNACGLYMKLHGVPRPLAMRKEGIQTRKRKPKNLNKSKTPAAPSGS-E 338
SP|P46152|Rat               TLWRRNAEGEPVCNACGLYMKLHGVPRPLAMRKEGIQTRKRKPKNLNKSKTPAGPPG--E 336
SP|Q0Q0E4|Dog               TLWRRNAEGEPVCNACGLYMKLHGVPRPLAMRKEGIQTRKRKPKNLNKSKTPAGPSGG-E 338
TR|I3LUZ9|Pig               TLWRRNAEGEPVCNACGLYMKLHGVPRPLAMRKEGIQTRKRKPKNLNKSKTSAGPSGS-E 338
SP|P43691|Chicken           TLWRRNAEGEPVCNACGLYMKLHGVPRPLAMRKEGIQTRKRKPKNLNKTKTPAGPSSS-E 278
TR|A0A2I3SFW5|Chimpanzee    TLWRRNAEGEPVCNACGLYMKLHGVPRPLAMRKEGIQTRKRKPKNLNKSKTPAAPSGS-E 339
TR|M3WLT5|Cat               TLWRRNAEGEPVCNACGLYMKLHGVPRPLAMRKEGIQTRKRKPKNLNKSKTPAGPSGS-E 338
TR|F1N551|Bovine            TLWRRNAEGEPVCNACGLYMKLHGVPRPLAMRKEGIQTRKRKPKNLNKSKTPAGPSGS-E 338
TR|F6QQ97|Rhesus macaque    TLWRRNAEGEPVCNACGLYMKLHGVPRPLAMRKEGIQTRKRKPKNLNKSKTPAGPAGS-E 309
TR|H9G5S5|Am. chameleon     TLWRRNAEGEPVCNACGLYMKLHGVPRPLAMRKEGIQTRKRKPKNVNKSKTSAGSSSNAE 313
TR|F7CJW8|Horse             TLWRRNAEGEPVCNACGLYMKLHGVPRPLAMRKEGIQTRKRKPKNLNKSKTPAGPSGS-E 338
TR|F7B924|Opossum           TLWRRNAEGEPVCNACGLYMKLHGVPRPLAMRKEGIQTRKRKPKNLNKAKTTTGPSGS-E 349
TR|H2PPJ2|Sum. orangutan    TLWRRNAEGEPVCNACGLYMKLHGVPRPLAMRKEGIQTRKRKPKNLNKSKTSAAPSGS-E 338
TR|G1SH13|Rabit             TLWRRNAEGEPVCNACGLYMKLHGVPRPLAMRKEGIQTRKRKPKNLNKSKTPAGAAGS-E 339
TR|M3XRU8|Ferret            TLWRRNAEGEPVCNACGLYMKLHGVPRPLAMRKEGIQTRKRKPKNLNKSKTPAGPSGG-E 338
TR|A0A096N2A9|Olive baboon  TLWRRNAEGEPVCNACGLYMKLHGVPRPLAMRKEGIQTRKRKPKNLNKSKTPAGPAGS-E 338
TR|A0A0D9RWJ0|Green monkey  TLWRRNAEGEPVCNACGLYMKLHGVPRPLAMRKEGIQTRKRKPKNLNKSKTPAGPAGS-E 338
TR|G1LHY0|Giant panda       TLWRRNAEGEPVCNACGLYMKLHGVPRPLAMRKEGIQTRKRKPKNLNKSKTPAGPSGS-E 344
TR|A0A3Q0CU09|Golden hamster TLWRRNAEGEPVCNACGLYMKLHGVPRPLAMRKEGIQTRKRKPKNLNKSKASAGPPG--E 335
TR|H0ZSU3|Zebra finch       TLWRRNAEGEPVCNACGLYMKLHGVPRPLAMRKEGIQTRKRKPKNLNKTKAPAGPSSS-E 278
TR|A0A452E0B6|Goat          TLWRRNAEGEPVCNACGLYMKLHGVPRPLAMRKEGIQTRKRKPKNLNKSKTPAGPSGS-E 338
TR|W5Q0U8|Sheep             TLWRRNAEGEPVCNACGLYMKLHGVPRPLAMRKEGIQTRKRKPKNLNKSKTPAGPSGS-E 242
TR|A0A2K5V779|Cyn. monkey   TLWRRNAEGEPVCNACGLYMKLHGVPRPLAMRKEGIQTRKRKPKNLNKSKTPAGPAGS-E 321
TR|G3SQY9|African elephant  TLWRRNAEGEPVCNACGLYMKLHGVPRPLAMRKEGIQTRKRKPKNLNKSKTPAGPSGS-E 339
                            ****** .*********** *** * ***:*:*:*****:** .  .:
```

Supplementary Figure S10. High sequence conservation in zinc finger domain of GATA4 among different species. Human and other mammals are unable to regenerate cardiomyocytes after birth and express an arginine (hR310, purple) at the C-terminal tail of the zinc finger. However, species with regenerative capacity, including Zebrafish (Q09JY7), Eastern newt (F2W888), Bat star (Q6XZF5), Sea cucumber (A0A2G8JQ98), African clawed frog (Q91677) and Hydra vulgaris (T2MH05) have consistent expression of lysine at the same position (green). Conserved residues responsible for C_4-

coordination of zinc fingers are highlighted with grey colour. In the bottom row, the alignment results are represented as follows: The asterisk (*) indicates a single and fully conserved residue. A colon (:) indicates conservation between groups of strongly similar properties. A period (.) indicates conservation between groups with weakly similar properties. A number at the end of the line indicates the running number of the last amino acid of the respective sequence. Protein sequences were downloaded from UniProt Knowledgebase (UniProtKB) which contains two separate sections; UniProtKB/Swiss-Prot (SP, manually annotated) and UniProtKB/TrEMBL (TR, computationally annotated). Sequences were aligned by using Clustal Omega (European Bioinformatics Institute, EMBL-EBI).

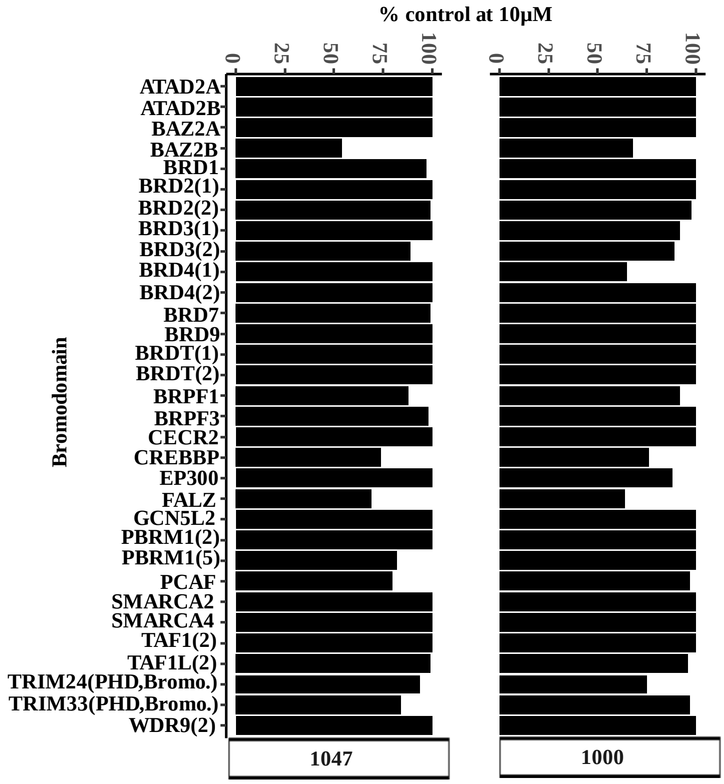

Supplementary Figure S11. BromoMAX assay shows no significant perturbation of bromodomain proteins in cell-free assays by compounds 3i-1000 and 3i-1047 at 10 µM, indicating that the acetyl-lysine like domain within the compounds do not bind directly to bromodomains.

Supplementary Tables

Supplementary Table S1. Taqman assays used for the characterization of embryoid bodies from chemically-induced differentiation experiments.

Gene	Assay ID
Tbx5	Mm00803521_m1
Irx4	Mm00502170_m1
Hey2	Mm00469280_m1
Kcne1	Mm01215533_m1
Myl2	Mm00440384_m1
Nr2f2	Mm00772789_m1
Myl7	Mm00491655_m1
Gja5	Mm01265686_m1
Sln	Mm00481536_m1
Pitx2	Mm01316994_m1
Tnnt2	Mm01290256_m1
Kit	Mm00445212_m1
Isl1	Mm00627860_m1
Kdr	Mm01222421_m1
pdgfra	Mm00440701_m1
Nkx-2-5	Mm00657783_m1
Gata4	Mm00484689_m1
T	Mm00436877_m1
Pecam1	Mm01242576_m1
Myh11	Mm00443013_m1
Acta2	Mm01546133_m1
Thy1	Mm00493681_m1
Cdh2	Mm01162497_m1
Vim	Mm01333430_m1
Axin2	Mm00443610_m1
Alcam	Mm00711623_m1
Cyp26a1	Mm00514486_m1
Cyp26c1	Mm03412454_m1
Aldh1a2	Mm00501306_m1
actb	Mm00607939_s1
Nppa	Rn00664637_g1
Nppb	Mm01255770_g1

Supplementary Table S2. Structural derivatives of GATA-targeted compounds [3, 5] examined for stage-specific activation of atrial and ventricular reporter genes in differentiating pluripotent stem cells.

Code	Molecular structure	Molecular weight	GATA4-NKX2-5 synergy at 10 µM (% of control)*	Supplier (code)	Reference
**3i-0595**	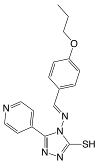	339 Da	< 90%	Pharmatory Oy Oulu, Finland	Välimäki *et al.* 2017
**3i-0662**	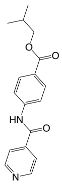	298 Da	< 90%	Chembridge (6125548)	Välimäki *et al.* 2017
**3i-0723**	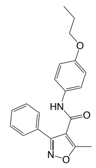	336 Da	90%–110%	Chembridge (7703003)	Välimäki *et al.* 2017
**3i-0777**	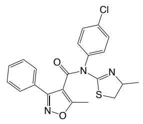	411 Da	170%	Pharmatory Oy Oulu, Finland	Välimäki *et al.* 2017 Jumppanen *et al.* 2019
**3i-1000**	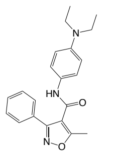	349 Da	50%	Pharmatory Oy Oulu, Finland	Välimäki *et al.* 2017 Jumppanen *et al.* 2019
**3i-1008**	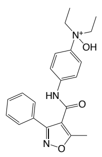	356 Da	94%	Faculty of Pharmacy Univ. of Helsinki	Jumppanen *et al.* 2019
**3i-1012**	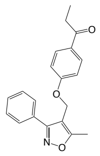	321 Da	76%	Faculty of Pharmacy Univ. of Helsinki	Jumppanen *et al.* 2019
**3i-1013**	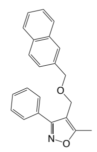	329 Da	91%	Faculty of Pharmacy Univ. of Helsinki	Jumppanen *et al.* 2019
**3i-1021**	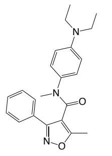	363 Da	97%	Faculty of Pharmacy Univ. of Helsinki	Jumppanen *et al.* 2019
**3i-1029**	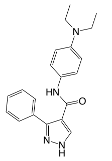	334 Da	93%	Faculty of Pharmacy Univ. of Helsinki	Jumppanen *et al.* 2019

**3i-1037**	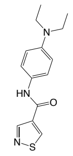	275 Da	27%	Faculty of Pharmacy Univ. of Helsinki	Jumppanen *et al.* 2019
**3i-1040**	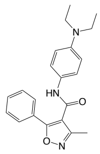	349 Da	29%	Faculty of Pharmacy Univ. of Helsinki	Jumppanen *et al.* 2019
**3i-1043**	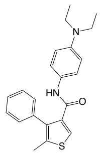	364 Da	33%	Faculty of Pharmacy Univ. of Helsinki	Jumppanen *et al.* 2019
**3i-1044**	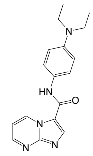	309 Da	144%	Faculty of Pharmacy Univ. of Helsinki	Jumppanen *et al.* 2019
**3i-1047**	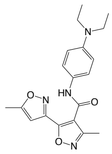	354 Da	83%	Faculty of Pharmacy Univ. of Helsinki	Jumppanen *et al.* 2019
**3i-1048**	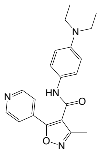	350 Da	64%	Faculty of Pharmacy Univ. of Helsinki	Jumppanen *et al.* 2019
**3i-1103**	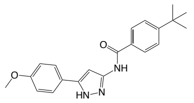	349 Da	49%	Faculty of Pharmacy Univ. of Helsinki	Jumppanen *et al.* 2019
**3i-1148**	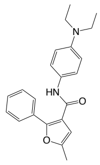	348 Da	35%	Faculty of Pharmacy Univ. of Helsinki	Jumppanen *et al.* 2019
**3i-1165**	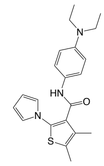	367 Da	79%	Faculty of Pharmacy Univ. of Helsinki	Jumppanen *et al.* 2019
**3i-1194**	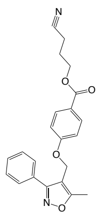	376 Da	52%	Faculty of Pharmacy Univ. of Helsinki	Jumppanen *et al.* 2019

**3i-1212**	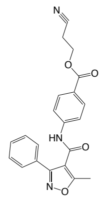	375 Da	104%	Faculty of Pharmacy<br>Univ. of Helsinki	Jumppanen *et al.* 2019
**3i-1228**	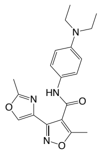	354 Da	104%	Faculty of Pharmacy<br>Univ. of Helsinki	Jumppanen *et al.* 2019
**3i-1229**	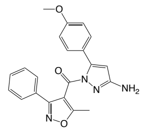	374 Da	72%	Faculty of Pharmacy<br>Univ. of Helsinki	Jumppanen *et al.* 2019
**3i-1233**	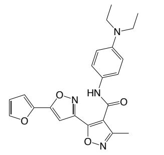	406 Da	100%	Faculty of Pharmacy<br>Univ. of Helsinki	Jumppanen *et al.* 2019
**3i-1234**	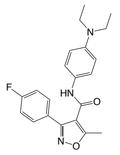	367 Da	44%	Faculty of Pharmacy<br>Univ. of Helsinki	Jumppanen *et al.* 2019
**3i-1235**	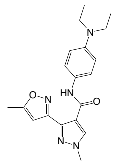	353 Da	99%	Faculty of Pharmacy<br>Univ. of Helsinki	Jumppanen *et al.* 2019
**3i-1236**	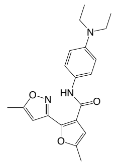	353 Da	104%	Faculty of Pharmacy<br>Univ. of Helsinki	Jumppanen *et al.* 2019
**3i-1237**	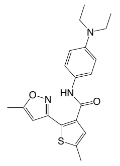	369 Da	85%	Faculty of Pharmacy<br>Univ. of Helsinki	Jumppanen *et al.* 2019
**3i-1238**	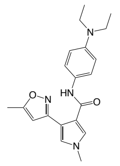	352 Da	111%	Faculty of Pharmacy<br>Univ. of Helsinki	Jumppanen *et al.* 2019
**3i-2042**	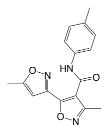	297 Da	86%	Maybridge<br>(SPB03214)	Jumppanen *et al.* 2019
**3i-2043**	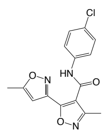	317 Da	88%	Maybridge<br>(SPB03211)	Jumppanen *et al.* 2019

**3i-2045**	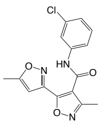	317 Da	82%	Maybridge (SPB03213)	Jumppanen *et al.* 2019

* Compound classification based on GATA4-NKX2-5 synergy; < 90%, inhibitory compounds, 90%–110% inactive compounds, and > 110% agonistic compounds.

Supplementary Table S3. Gene ontology (GO) enrichment analysis of CRAPome-filtered BioID-results for GATA4 (320 identified proteins) determines the most abundant functional associations.

	Homo sapiens	Client Text Box Input				
GO molecular function complete	#	#	expected	Fold Enrichment	+/-	P value
histone methyltransferase activity (H3-K4 specific)	16	6	.24	24.51	+	1.91E-03
histone-lysine N-methyltransferase activity	42	8	.64	12.45	+	2.12E-03
lysine-acetylated histone binding	20	6	.31	19.61	+	5.61E-03
histone binding	204	26	3.12	8.33	+	3.97E-12
histone acetyltransferase activity	61	16	.93	17.14	+	8.14E-11
peptide-lysine-N-acetyltransferase activity	63	16	.96	16.60	+	1.25E-10
histone deacetylase activity	28	6	.43	14.01	+	2.96E-02
repressing transcription factor binding	73	14	1.12	12.54	+	1.32E-07
DNA-binding transcription factor binding	368	44	5.63	7.82	+	3.07E-21
RNA polymerase II complex binding	37	7	.57	12.37	+	1.10E-02
nuclear receptor transcription coactivator activity	69	13	1.06	12.31	+	7.99E-07
transcription coactivator activity	339	51	5.19	9.83	+	2.15E-29
androgen receptor binding	44	8	.67	11.88	+	2.89E-03
nuclear receptor binding	114	17	1.74	9.75	+	3.89E-08
RNA polymerase II transcription factor binding	59	10	.90	11.08	+	2.28E-04
p53 binding	68	11	1.04	10.57	+	7.82E-05
transcription corepressor activity	248	40	3.79	10.54	+	2.02E-23
promoter-specific chromatin binding	58	9	.89	10.14	+	2.00E-03
chromatin binding	568	74	8.69	8.52	+	1.38E-40
nucleosome binding	67	9	1.03	8.78	+	5.91E-03
methylated histone binding	66	8	1.01	7.92	+	4.35E-02
histone deacetylase binding	114	13	1.74	7.45	+	1.86E-04
beta-catenin binding	88	9	1.35	6.68	+	4.50E-02
RNA polymerase II cis-regulatory region sequence-specific DNA binding	609	57	9.32	6.12	+	1.94E-23
cis-regulatory region sequence-specific DNA binding	633	60	9.68	6.20	+	3.90E-25
RNA polymerase II regulatory region sequence-specific DNA binding	847	72	12.96	5.56	+	3.35E-28
DNA-binding transcription repressor activity, RNA polymerase II-specific	256	21	3.92	5.36	+	4.35E-06
DNA-binding transcription factor activity, RNA polymerase II-specific	1033	82	15.80	5.19	+	7.90E-31
DNA-binding transcription repressor activity	257	21	3.93	5.34	+	4.64E-06
helicase activity	160	13	2.45	5.31	+	6.61E-03
DNA-binding transcription activator activity, RNA polymerase II-specific	441	31	6.75	4.59	+	2.00E-08
DNA-binding transcription activator activity	444	31	6.79	4.56	+	2.36E-08
protein domain specific binding	710	29	10.86	2.67	+	7.91E-03
zinc ion binding	841	34	12.87	2.64	+	1.31E-03
metal ion binding	4254	108	65.08	1.66	+	7.46E-05
Unclassified	3169	10	48.48	.21	-	0.00E00

Supplementary Table S4. Gene ontology (GO) enrichment analysis of CRAPome-filtered BioID-results for NKX2-5 (359 identified proteins) determines the most abundant functional associations.

	Homo sapiens	Client Text Box Input				
GO molecular function complete	#	#	expected	Fold Enrichment	+/-	P value
SUMO ligase activity	9	5	.15	32.45	+	6.53E-03
histone methyltransferase activity (H3-K4 specific)	16	6	.27	21.90	+	3.64E-03
histone-lysine N-methyltransferase activity	42	8	.72	11.12	+	4.86E-03
histone deacetylase activity	28	7	.48	14.60	+	4.55E-03
protein deacetylase activity	29	7	.50	14.10	+	5.57E-03
RNA polymerase II transcription factor binding	59	14	1.01	13.86	+	4.82E-08
transcription factor binding	692	79	11.85	6.67	+	4.54E-36
histone acetyltransferase activity	61	13	1.04	12.45	+	8.18E-07
peptide-lysine-N-acetyltransferase activity	63	13	1.08	12.05	+	1.16E-06
nuclear receptor transcription coactivator activity	69	14	1.18	11.85	+	2.96E-07
transcription coactivator activity	339	53	5.80	9.13	+	6.40E-29
RNA polymerase II repressing transcription factor binding	35	7	.60	11.68	+	1.65E-02
RNA polymerase II-specific DNA-binding transcription factor binding	286	34	4.90	6.94	+	3.15E-14
repressing transcription factor binding	73	15	1.25	12.00	+	5.40E-08
transcription corepressor activity	248	47	4.25	11.07	+	1.40E-28
RNA polymerase II complex binding	37	7	.63	11.05	+	2.28E-02
androgen receptor binding	44	8	.75	10.62	+	6.61E-03
nuclear receptor binding	114	18	1.95	9.22	+	2.60E-08
steroid hormone receptor binding	93	13	1.59	8.16	+	7.66E-05
promoter-specific chromatin binding	58	9	.99	9.06	+	4.99E-03
chromatin binding	568	76	9.73	7.81	+	7.11E-39
histone deacetylase binding	114	17	1.95	8.71	+	2.21E-07
p53 binding	68	10	1.16	8.59	+	2.06E-03
nucleosome binding	67	9	1.15	7.85	+	1.46E-02
histone binding	204	24	3.49	6.87	+	2.77E-09
beta-catenin binding	88	10	1.51	6.64	+	1.71E-02
activating transcription factor binding	90	10	1.54	6.49	+	2.05E-02
RNA polymerase II cis-regulatory region sequence-specific DNA binding	609	64	10.43	6.14	+	1.64E-26
cis-regulatory region sequence-specific DNA binding	633	68	10.84	6.27	+	6.09E-29
RNA polymerase II regulatory region sequence-specific DNA binding	847	83	14.50	5.72	+	1.26E-33
modification-dependent protein binding	149	13	2.55	5.10	+	1.07E-02
DNA-binding transcription activator activity, RNA polymerase II-specific	441	35	7.55	4.64	+	7.95E-10
DNA-binding transcription activator activity	444	35	7.60	4.60	+	9.54E-10
DNA-binding transcription factor activity, RNA polymerase II-specific	1033	90	17.69	5.09	+	3.43E-33
DNA-binding transcription repressor activity, RNA polymerase II-specific	256	20	4.38	4.56	+	1.47E-04
DNA-binding transcription repressor activity	257	20	4.40	4.55	+	1.56E-04
Unclassified	3169	12	54.26	.22	-	0.00E00

Supplementary Table S5. Summary of differentially expressed genes following 30 and 120 minute treatments with GATA4-targeted compound 3i-1000 versus DMSO control in neonatal rat ventricular cardiomyocytes.

Transcript ID	Gene Symbol	30 min 3i-1000 vs 30 min DMSO (logFC)	120 min 3i-1000 vs 120 min DMSO (logFC)	30 min DMSO	30 min 3i-1000	120 min DMSO (RPKM)	120 min 3i-1000 (RPKM)
NM_172075	Thop1	-0,87	-0,72	6,22	5,31	6,1	5,38
NM_001105771	Mrps34	-1,04	-0,67	6,27	5,19	5,97	5,3
NM_001109670	Slc52a2	-0,77	-0,7	5,89	5,07	5,7	5,03
NM_001013886	Tubb2b	-0,72	-0,73	5,51	4,76	5,62	4,92
NM_001017377	Paqr4	-0,68	-0,67	5,45	4,71	5,44	4,76
NM_212490	Atp6v1g2	-1,14	-0,85	5,75	4,6	5,41	4,59
NM_172035	Fzd2	-0,67	-0,86	6,86	6,14	6,54	5,67
NM_031349	Aplnr	-0,95	-0,67	6,83	5,83	6,59	5,9
NM_001276434	Hmgn5	0,99	1,04	4,89	5,79	4,56	5,51
NM_001134706	Hmgn5b	0,99	1,04	4,88	5,79	4,54	5,53
NM_001107253	Kctd6	0,72	0,68	5,57	6,2	4,89	5,51
NM_019361	Arc	0,71	2,18	2,92	3,49	3,74	5,72
NM_017178	Bmp2	0,67	0,72	6,31	6,9	6,68	7,37
NM_031628	Nr4a3	1,03	2,04	5,06	6,02	6,85	8,85
NM_024388	Nr4a1	1,09	1,33	6,74	7,75	9,54	10,84

Supplementary Methods

2.1 Plasmids

The rat BNP minimal promoter together with −90 tandem GATA-site containing luciferase reporter vector (NP112) has been described previously [2]. The luciferase reporter vector pGL3-3xHA containing three high affinity binding sites for NKX2-5 (3xHA-NKX2-5) in front of a rat albumin minimal promoter has been described earlier [4]. The plasmid expressing the mouse GATA4 (pMT2-GATA4) and the empty pMT2 plasmid were gifts from D. B. Wilson (Department of Pediatrics, St. Louis Children's Hospital) [1]. The plasmid expressing mouse NKX2-5 (pMT2-NKX2-5) has been described earlier [4].

2.2 Immunoblotting of GATA4

GATA4 protein was examined in mESC-derived EBs collected on D5 and D12 of differentiation with compound treatments. Cells were trypsinized and the pellets were lysed into 1% SDS in 50 mM Tris-HCl, pH 7.4 by using 25G needle. The protein concentration was measured using Pierce BCA Protein Assay Kit (Thermo Scientific). Samples were prepared into Laemmli buffer and 80 µg or 30 µg of protein was loaded onto 10% SDS-PAGE gel. For overexpression studies in HEK293 cells, a plasmid containing a tetO-GATA4-V5 cassette was obtained from Addgene (#46030). Additionally, a reverse tetracycline transactivator-encoding plasmid (Addgene #19780) was obtained for transactivation of the tetO promoter. HEK293 cells ($0.5x10^6$) were transfected the day after plating with 2.5 µg plasmid using Lipofectamine 3000 transfection reagent (Thermo Fisher Scientific). Cells were cultured in the presence of doxycycline (2 µg/mL) to induce transcription from the tetO promoter. Cells were collected directly into 200 µL 4× Laemmli buffer with 2-mercaptoethanol and boiled for 5 minutes. From the crude cell lysate, a sample was diluted 1/5 and further 1/25 with 1× Laemmli buffer. A 10 µl sample from each dilution: the crude, 1/5 and 1/25 was run on 10% SDS-PAGE and transferred to a nitrocellulose membrane. For membrane blocking and antibody dilutions, 5% BSA in tris-buffered saline (TBS)-Tween (0.1%) was used. Antibodies used for immunoblotting include anti-GATA4 (Santa Cruz Biotechnology, sc-9053) 1:1000, anti-V5 (Cell Signaling Technology, #13202) 1:1000, anti-β-actin (Cell Signaling Technology, #4967) 1:1000 and goat antirabbit immunoglobulin G (IgG) 1:2000 (#7074). For a second immunoblotting, the membrane was stripped for 30 min at +60 °C in stripping buffer (60 mM Tris pH 6.8, 2% SDS and 100 mM β-mercaptoethanol). SuperSignal West Pico Chemiluminescent Substrate (34080, ThermoFisher Scientific) was used to visualize the immune complexes and chemiluminescence was detected and digitalized with a Luminescent Image Analyzer LAS-3000 (Fujifilm).

2.3 Cardiomyocyte cell culture

The neonatal rat ventricular myocytes (NRVM) were isolated from 2- to 4-day-old Sprague Dawley rats, as described earlier [5]. Rat pups were acquired from Oulu Laboratory Animal Centre (University of Oulu) with an internal use license. All animal experiments were conducted in accordance with the guidelines laid for protection of animals used for scientific purposes by directive 2010/63/EU, and National Institutes of Health (NIH). After the enrichment of myocytes by preplating, the cells were counted and seeded on a 10 cm cell culture dish with 10 M cells/dish and cultured for 18−20 h with Dulbecco's modified Eagle medium (DMEM)/F12 culture medium containing 2.5 mM L-glutamine, supplemented with 100 IU/ mL penicillin−streptomycin and 10% fetal bovine serum (all from Gibco, Thermo Scientific). Prior to exposure to compounds, the cells were cultured for 24 h in serum free media (CSFM; DMEM/F12, 2.5 mg/mL bovine serum albumin, 1 µM insulin, 2.5 mM L-glutamine, 32 nM selenium, 2.8 mM sodium pyruvate, 5.64 µg/mL transferrin, 1 nM T3, and 100 IU/mL PS). The compound 3i-1000 (3 µM) or the vehicle (0.1% DMSO) were added to cells in CSFM media for 30 or 120 minutes and the nuclei were isolated for GRO-seq.

2.4 GRO-seq nuclei isolation

During the entire isolation protocol the samples were kept on ice or centrifugations performed at 4 °C. The used buffers were also pre-cooled to ice cold. The NRVM were collected by using trypsin, centrifuged at 400 *g* for 5 min and re-suspended into PBS. The cells were washed twice with PBS and resuspended into 10 ml of Swelling buffer (10 mM Tris-HCl pH7.5, 2 mM $MgCl_2$, 3 mM $CaCl_2$ in ultrapure water) supplemented with 2 U/ml SUPERase-In RNase Inhibitor (Ambion, AM2696) and incubated for 5 min. The cells were collected by centrifugation at 400 *g* for 10 min and re-suspended in 500 µl of Swelling buffer with 10% glycerol and 4 U/ml SUPERase-In. The sample was vortexed slowly (~800 rpm, VortexGenie2 speed 1-3) while adding drop by drop 500 µl of Swelling buffer with 10% glycerol, 1% Igepal and 2 U/ml SUPERase-In and incubated for 5 min. The sample volume was adjusted to 10 ml with Lysis buffer (Swelling buffer with 0.5% Igepal, 10% glycerol and 2 U/ml SUPERase-In) and centrifuged at 600 *g* for 5 min. Nuclei were washed once more with Lysis buffer and collected by centrifugation as in previous step. The nuclei were re-suspended in 1 ml of Freezing buffer (40% glycerol, 5 mM $MgCl_2$, 0.1 mM EDTA, 50 mM Tris-HCl pH 8.3, 2 U/ml SUPERase-In in ultrapure water) and counted with a hemocytometer.

2.5 Reporter gene assays

COS-1 cells were cultured in Dulbecco's modified Eagle's medium (Sigma-Aldrich/Gibco) containing 10% fetal bovine serum (Gibco) and 1% penicillin−streptomycin (Sigma-Aldrich) (100 U/mL and 0.1 mg/mL, respectively). For luciferase assays, COS-1 cells were seeded at 10 000 cells/well in Isoplate-96 microplates (PerkinElmer, Turku, Finland) and grown overnight. In GATA reporter assays, the cells were transfected with NP112 100 ng/well and pMT2-GATA4 25 ng/well. In NKX2-5-GATA4 synergy reporter assays, the cells were transfected with 3xHA-NKX2-5 100 ng/ well and equal amounts of pMT2-GATA4 and pMT2-NKX2-5 total 50 ng/well. To verify the synergistic effect, the cells were transfected with pMT2-GATA4 or pMT2-NKX2-5 25 ng/ well, and the total DNA concentration was adjusted to 50 ng/well with empty pMT2 plasmid. The ratio of DNA/transfection reagent (Fugene 6, Promega) was 1:3 in all assays. The transfections were performed in serum and antibiotic-free media for 6 h. The cells were treated with compounds or vehicle (0.1% DMSO). The second compound was added to cells 30 min after the first compound. In case of only one compound, vehicle was added at first and the compound 30 min later. After 24 hours, the luminescence was measured using neolite Reporter Gene Assay System (PerkinElmer, Turku, Finland) and Victor2 plate reader (PerkinElmer, Turku, Finland).

References

1. Arceci RJ, King AA, Simon MC, Orkin SH, Wilson DB (1993) Mouse GATA-4: a retinoic acid-inducible GATA-binding transcription factor expressed in endodermally derived tissues and heart. Mol Cell Biol 13:2235–2246
2. Grepin C, Dagnino L, Robitaille L, Haberstroh L, Antakly T, Nemer M (1994) A hormone-encoding gene identifies a pathway for cardiac but not skeletal muscle gene transcription. Mol Cell Biol 14:3115–3129
3. Jumppanen M, Kinnunen SM, Välimäki MJ, Talman V, Auno S, Bruun T, Boije Af Gennäs G, Xhaard H, Aumüller IB, Ruskoaho H, Yli-Kauhaluoma J (2019) Synthesis, identification, and structure-activity relationship analysis of GATA4 and NKX2-5 protein-protein Interaction Modulators. J Med Chem 62:8284–8310. doi: 10.1021/acs.jmedchem.9b01086
4. Kinnunen S, Välimäki M, Tölli M, Wohlfahrt G, Darwich R, Komati H, Nemer M, Ruskoaho H (2015) Nuclear receptor-like structure and interaction of congenital heart disease-Associated factors GATA4 and NKX2-5. PLoS One 10:e0144145. doi: 10.1371/journal.pone.0144145
5. Välimäki MJ, Tölli MA, Kinnunen SM, Aro J, Serpi R, Pohjolainen L, Talman V, Poso A,

Ruskoaho HJ (2017) Discovery of Small Molecules Targeting the Synergy of Cardiac Transcription Factors GATA4 and NKX2-5. J Med Chem 60:7781–7798. doi: 10.1021/acs.jmedchem.7b00816
